# *Panax notoginseng* Saponins Accelerate Diabetic Wound Repair by Minimizing Mitochondrial Dysfunction and Inhibiting Pyroptosis of Macrophage via Regulating Ephx2/11,12‐EET Axis

**DOI:** 10.1002/fsn3.72494

**Published:** 2026-10-16

**Authors:** Nan Chen, Bo Zhu, Cai‐hua Sun, Tong Hou

**Affiliations:** ^1^ The First Affiliated Hospital of Zhejiang Chinese Medical University (Zhejiang Provincial Hospital of Chinese Medicine) Hangzhou P. R. China; ^2^ State Key Laboratory for Quality Ensurance and Sustainable Use of Dao‐di Herbs Beijng P. R. China; ^3^ School of Public Health Zhejiang Chinese Medical University Hangzhou P. R. China

**Keywords:** diabetic wound, Ephx2/11,12‐EET axis, mitochondrial homeostasis, *Panax notoginseng* saponins, pyroptosis

## Abstract

Impaired diabetic wound (DW) healing is mainly driven by excessive inflammatory response, while *Panax notoginseng* saponins (PNS), the core bioactive components of *Panax notoginseng*, show pro‐healing potential in diabetic ulcers with unclear mechanisms. Here, we established a streptozotocin‐induced DW mouse model, and combined untargeted metabolomics with in vitro and in vivo validations to decipher PNS's therapeutic mechanism and active ingredients. In vivo, PNS dose‐dependently accelerated wound closure, enhanced collagen deposition, and improved glucose tolerance and insulin sensitivity in diabetic mice, without affecting body weight or random blood glucose. Mechanistically, PNS inhibited Ephx2 expression (at both mRNA and protein levels) in macrophages to upregulate 11,12‐EET, thus mitigating wound inflammation, NLRP3 inflammasome‐mediated pyroptosis, and mitochondrial dysfunction. In vitro, the protective effects of PNS on macrophage mitochondrial function and pyroptosis were not enhanced by Ephx2 inhibitor TPPU, but completely abolished by 11,12‐EET antagonist 14,15‐EEZE. Furthermore, five major saponins (R1, Rg1, Re, Rb1, Rd) from PNS were identified as active ingredients, all exerting pro‐healing effects in DW mice. Conclusively, PNS accelerates DW healing via regulating the macrophage Ephx2/11,12‐EET axis to preserve mitochondrial homeostasis and inhibit pyroptosis, supporting its therapeutic potential for diabetic foot ulcers.

## Graphical abstract

*Panax notoginseng* saponins (PNS) accelerate diabetic wound healing via targeting the Ephx2/11,12‐EET axis in macrophages. PNS inhibits Ephx2 expression to elevate 11,12‐EET levels, which restores mitochondrial homeostasis by regulating mitochondrial dynamic proteins, reduces mtROS production, and subsequently suppresses NLRP3 inflammasome‐mediated pyroptosis, ultimately alleviating excessive inflammation and promoting tissue repair in diabetic wounds.

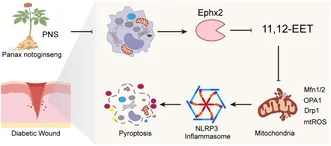

**Abbreviations**
11,12‐EET: 11,12‐epoxyeicosatrienoic acid
DFU: diabetic foot ulcer
Drp1: dynamin‐related protein 1
DW: diabetic wound
GTT: glucose tolerance test
HL: high glucose and lipopolysaccharide
ITT: insulin tolerance test
LPS: lipopolysaccharide
Mfn1/2: mitochondrial fusion protein 1/2
mtROS: mitochondrial reactive oxygen species
OPA1: optic atrophy1
PNS: *Panax notoginseng* saponins
ROS: reactive oxygen species
sEH: soluble epoxide hydrolase
STZ: streptozotocin

## 1 Introduction

Diabetes mellitus is a metabolic disorder primarily characterized by high blood glucose levels over a prolonged period (Packer et al. 2025). It is prevalent in the population and cannot be completely cured, posing a significant threat to patients' health (Chen et al. 2023). Currently, diabetes mellitus affects approximately 828 million adults globally (Chi et al. 2024). Chronic hyperglycemia frequently impairs wound healing mechanisms, which causes diabetic foot ulcers (DFUs). A limb is amputated due to DFU every 20 s, and the associated 5‐year mortality rate is nearly 30% (Armstrong et al. 2023; Zhang et al. 2023). Current DFU therapies primarily focus on glycemic control and wound healing; however, these strategies often fail to meet clinical requirements (Kumbhar and Bhatia 2024). Consequently, developing more effective and targeted pharmacological interventions to accelerate diabetic wound healing is a top objective.

Superficially, the difficulty in achieving complete healing or delayed healing of wounds in DFU patients is attributed to metabolic abnormalities caused by persistent hyperglycemia (Yang et al. 2025). Abnormal metabolites are the products of imbalanced metabolic reactions within the organism and often mark the onset of downstream inflammatory responses (Ryan et al. 2024). Mechanistically, macrophages are central regulators of inflammation and tissue repair (DeBerge et al. 2025; Dutta et al. 2025). Their polarization into pro‐inflammatory (M1) or pro‐reparative (M2) subtypes alters the wound microenvironment, which is a key pathological feature of non‐healing DFUs (Luo et al. 2025). Pyroptosis, due to its characteristic excessive inflammatory signaling, impedes the healing of diabetic wounds (Yang et al. 2023), a phenomenon particularly pronounced in M1 macrophages. Recent research indicates that the occurrence of pyroptosis is attributed to mitochondrial homeostasis imbalance, specifically abnormalities in mitochondrial fission (mediated by dynamin‐related protein 1 (Drp1)) and fusion (mediated by mitofusins 1/2 (Mfn1/2) and optic atrophy 1 (OPA1)) (Gordaliza‐Alaguero et al. 2025; Miya et al. 2025). Disrupted mitochondrial dynamics cause membrane potential collapse, mitochondrial reactive oxygen species (mtROS) overproduction, and leakage, which activates the NLRP3 inflammasome (Bellut et al. 2021). This subsequently triggers proteolytic activation of gasdermin D (GSDMD), that induce pyroptosis and secrete pro‐inflammatory cytokines, including interleukin‐1 beta (IL‐1β), IL‐6, and IL‐18 (Hou et al. 2024; Jafarzadeh et al. 2025). In addition, the clinical trials of the traditional Chinese medicine (TCM) ointment ON101 have confirmed that suppressing the inflammatory state of macrophages significantly promotes the healing of diabetic wounds (Chang et al. 2026). Therefore, identifying suitable resources from traditional Chinese medicine to improve the metabolic balance of wounds in DFU patients and combat pyroptosis occurring has become a current research focus.

Epoxyeicosatrienoic acids (EETs), including 11,12‐EET, are bioactive lipid mediators generated from arachidonic acid by cytochrome P450 (CYP) epoxygenases, particularly the CYP2C and CYP2J subfamilies (Zhao et al. 2017). EETs exert potent anti‐inflammatory and tissue‐reparative effects (Aghasizadeh et al. 2025; Waltl et al. 2026). However, the biological half‐life of EETs is tightly regulated by soluble epoxide hydrolase (sEH, encoded by the Ephx2 gene), which rapidly hydrolyzes EETs into their corresponding dihydroxyeicosatrienoic acids (DHETs), metabolites with substantially reduced biological activity (Marowsky et al. 2025). Accumulating evidence indicates that sEH expression and activity are upregulated in diabetic tissues, leading to accelerated EET degradation and a consequent deficit in these protective mediators, which has been implicated in the pathogenesis of diabetic vascular complications, impaired wound healing, and chronic inflammation (Frömel et al. 2022). In particular, it has been demonstrated that CYP epoxygenase expression and EET levels are significantly reduced in the wound granulation tissue of diabetic (ob/ob) mice, and that exogenous 11,12‐EET administration accelerates wound closure, whereas the EET antagonist 14,15‐EEZE impairs healing. These findings established the sEH/EET axis as a promising therapeutic target for diabetic wound repair (Zhao et al. 2017). Building on this rationale, we employed untargeted metabolomics to screen for differential metabolites in diabetic wound tissue before and after PNS treatment, which led to the identification of 11,12‐EET as a key metabolite restored by PNS, prompting us to investigate the Ephx2/11,12‐EET axis as the mechanistic focus of this study.

*Panax notoginseng* (*Burkill*) *F.H. Chen* (Araliaceae) is a medicinal and edible herb used in TCM to promote blood circulation and reduce swelling for more than 400 years (Mancuso 2024). Currently, Panax notoginseng has been demonstrated to exert effects across multiple metabolic pathways, including lipid metabolism, energy metabolism, inflammation‐related metabolites, and hormone metabolism (Zhu et al. 2025). Its primary bioactive components have been identified as *Panax notoginseng* saponins (PNS), a compound with anti‐inflammatory, antioxidant, immunomodulatory, and mitochondrial‐protective properties (Zhang, Yin, et al. 2025). Previously, Xueshuantong injection, primarily composed with PNS, extensively employed in clinical settings across China to treat vascular blockages caused by hyperglycemia. It has been applied in the treatment of DFU (Xing et al. 2019). Additionally, PNS have been proven to promote wound closure in hyperglycemic murine models (Cao et al. 2022). Notably, PNS possesses the capacity to suppress macrophage oxidative stress and inflammatory responses while also promoting ECM remodeling (Men et al. 2020; Wang et al. 2025). However, the mechanism by which PNS reduces wound pyroptosis through improved diabetic wound metabolites remains unknown.

Therefore, this study employs untargeted metabolomics to screen for differential metabolites in diabetic wound mice before and after treatment. The aim is to explore the effects of PNS on macrophage and elucidate its underlying mechanisms, thereby gaining a more comprehensive understanding of its potential value in the diabetic wound healing process.

## 2 Materials and Methods

### 2.1 Animal Experimental Design

#### 2.1.1 Part I: Diabetic Wound Model Establishment and PNS Treatment

Male C57BL/6 mice (8 weeks old) were obtained from Shanghai Laboratory Animal Co. Ltd. (No. SYXK 2021‐0012) and housed under standard conditions with *ad libitum* access to food and water. The model of diabetic mice was established by intraperitoneal injection of streptozotocin (STZ; 50 mg/(kg·day), Sigma‐Aldrich, MO, USA) for five consecutive days. Mice with random blood glucose (RBG) levels exceeding 17 mmol/L one week after the first STZ injection were considered diabetic. Under isoflurane anesthesia, a single full‐thickness excisional wound (6 mm diameter) was made on the dorsum of each mouse. This study included diabetic wound (DW) mice and non‐diabetic wound (NW) mice as a control (*n* = 8 per group). Afterward, DW mice were allocated into four groups (*n* = 8 per group): one vehicle‐treated group and three PNS‐treated groups orally administered PNS at low [25 mg/(kg·day)], medium [75 mg/(kg·day)], or high [225 mg/(kg·day)] doses. Highly purified PNS extracts (purity ≥ 80%) were supplied by Chengdu Must Pharmaceutical Inc. (A0760). The body weight and RBG were measured periodically for 14 days. Upon termination, whole blood, serum, and wound tissues were analyzed.

#### 2.1.2 Part II: Pharmacological Intervention Studies

Using the aforementioned DW protocol, six groups of male mice (*n* = 8 per group) were selected. NW and DW groups were administered the vehicle daily via oral gavage. The four intervention groups received daily treatments for 10 days as follows: PNS alone (100 mg/kg, p.o.), TPPU alone (a soluble epoxide hydrolase (sEH) inhibitor; 1 mg/kg, p.o.; MedChemExpress, Shanghai, China), a combination of PNS (100 mg/kg, p.o.) and TPPU (1 mg/kg, p.o.), or a combination of PNS (100 mg/kg, p.o.) and 14,15‐EEZE (an 11,12‐EET antagonist; 0.1 mg/kg, i.p.; Cayman, MI, USA).

#### 2.1.3 Part III: Identification of Pharmacologically Active Constituents

Eight groups of male mice (*n* = 8 per group) were established using the DW model. NW and DW groups were administered the vehicle via oral gavage. In accordance with previous research (Xie et al. 2024), treatment and positive control groups were administered 10 mg/kg (p.o.) of PNS constituents (notoginsenoside R1 (R1) or ginsenoside Rg1 (Rg1), Re (Re), Rb1(Rb1), or Rd. (Rd)) and metformin (200 mg/kg, p.o.), respectively. All treatments were administered once daily for 10 days.

### 2.2 Glucose Tolerance Test (GTT) and Insulin Tolerance Test (ITT)

Prior to GTT and ITT experiments, mice endured a 6‐h fast. Glucose (2 g/kg) and insulin (0.5 U/kg) were administered intraperitoneally. Blood glucose levels were tested at 0, 30, 60, 90, and 120 min after glucose or insulin injection.

### 2.3 Transmission Electron Microscope (TEM)

Freshly excised wound skin tissues were fixed in 2.5% glutaraldehyde prepared in 0.1 M phosphate buffer (pH 7.4) for at least 4 h at 4°C. The samples were sliced into 1 mm^3^ pieces, post‐fixed in 1% osmium tetroxide for 2 h at room temperature, and dehydrated with a graded series of acetone (50%, 70%, 90%, and 100%). Subsequently, the tissues were infiltrated and embedded in EPON 812 resin. Ultrathin sections (80 nm) were obtained using the Leica UC7 ultramicrotome, mounted on copper grids, and double‐stained with uranyl acetate (15 min) and lead citrate (5 min). Images were captured using a Hitachi HT7800 TEM with 120 kV accelerating voltage.

### 2.4 Histological Analysis

Skin tissues were fixed in 4% paraformaldehyde for 24 h at 4°C. After dehydration, the samples were embedded in paraffin and cut into 5 μm thick sections. Hematoxylin and eosin and Masson staining were conducted based on standard protocols. Epithelial gap, inflammatory cell infiltration, and collagen density (expressed as the percentage of stained area per field) were quantified using ImageJ‐based semi‐automated morphometric analysis.

### 2.5 Immunofluorescence Staining

Paraffin‐embedded skin sections were treated with 3% bovine serum albumin to block excess sites after thermal repair. For double immunofluorescence staining, sections were incubated overnight at 4°C with primary antibodies against F4/80 (a macrophage marker; 1:100; Santa Cruz, sc‐377009) in combination with either iNOS (an M1 marker; 1:100; ABclonal, A0312) or Arg1 (an M2 marker; 1:100; ABclonal, A1847). The following day, the sections were incubated with fluorescently conjugated secondary antibodies (diluted at 1:100) and DAPI staining solution. Images were acquired using a fluorescence microscope (Leica, DMIL).

### 2.6 Protein Extraction and Western Blotting

High‐purity proteins were obtained from skin wound tissues and cultured cells using RIPA lysis buffer (Beyotime, P0013B), separated using SDS‐PAGE, and transferred to polyvinylidene difluoride membranes. After blocking with 5% (w/v) non‐fat milk for 1 h at 24°C, the membranes were incubated overnight at 4°C with primary antibodies (all at 1:2000 dilution). The following day, the bands in the membrane were incubated with secondary antibodies (1:5000) and chemiluminescence substrate, followed by imaging in the Bio‐Rad ChemiDoc MP imaging system. Band intensities were quantified by densitometry using ImageJ software and normalized for β‐actin expression. Primary antibodies are provided in Table S1.

### 2.7 RNA Extraction and Quantitative Real‐Time PCR (qPCR)

Total RNA was extracted from wound tissues and cells using the TRIzol kit. Reverse transcription was performed with PrimeScript RT Master Mix. Relative mRNA expression was quantified using SYBR Green‐based qPCR following the manufacturer's instructions. β‐Actin was used as the home gene, and relative expression levels were calculated using the 2^−ΔΔCt^ method. Primer sequences are provided in Table S2.

### 2.8 Untargeted Metabolomics

After homogenizing 100 mg of wound tissue, 80% methanol/water (v/v) was added for pretreatment. The mixture was centrifuged at 15,000 *g* for 20 min at 4°C, and the supernatant was diluted to 53%. The sample was recentrifuged under the same conditions to obtain the experimental sample. Quality control (QC) samples were prepared by pooling equal volumes from all experimental samples, and blank controls comprised 53% methanol/water (v/v).

Metabolomic profiling was conducted using an ultra‐high performance liquid chromatography (UHPLC) system (Thermo Fisher Scientific, Germany) coupled to a Q Exactive HF‐X hybrid quadrupole‐Orbitrap mass spectrometer (Thermo Fisher Scientific, Germany). Chromatographic separation was achieved on an Eclipse Plus C18 column (100 × 2.1 mm, 1.9 μm) with a mobile phase of 0.1% aqueous formic acid (A) and methanol (B) at 0.3 mL/min, followed by a 6‐min post‐run equilibration. Mass spectrometry parameters included a scan range of 100–1500 *m/z* in positive/negative ESI switching mode, 3.5 kV spray voltage, sheath gas (35 psi), auxiliary gas (10 L/min), capillary temperature (320°C), auxiliary gas heater (350°C), and data‐dependent MS/MS scans.

Raw UHPLC‐mass spectrometer (UHPLC‐MS) data were processed using Compound Discoverer software (version 3.3) for peak picking, alignment, deconvolution, and compound identification by searching against HMDB and METLIN databases. Data quality was assured through QC‐based normalization and batch effect correction.

Multivariate statistical analyses were performed using the metaX software, including principal component analysis (PCA) and orthogonal partial least squares‐discriminant analysis (OPLS‐DA). Differentially abundant metabolites between groups were identified based on the following criteria: |log2(fold change)| > 1.2, variable importance in projection score from the OPLS‐DA model > 1.0, and adjusted *p*‐value (Benjamini‐Hochberg FDR) < 0.05. Results were visualized using volcano plots.

### 2.9 Cell Culture and Experimental Design

The murine macrophage cell line RAW 264.7 (National Collection of Authenticated Cell Cultures, SCSP‐5036), a homogeneous macrophage‐lineage cell line, was cultured in Dulbecco's modified Eagle medium (DMEM; Gibco, 11885084) with 10% fetal bovine serum (FBS; Gibco, 10099141C) in a humidified incubator. The specific identification information is completed by F4/80 and CD11b; please refer to Figure S1 for details.

To establish a high glucose (HG)‐lipopolysaccharide (LPS) (HL) model in vitro to mimic the chronic inflammatory milieu of diabetic wounds (Zhong et al. 2026), RAW 264.7 cells were stimulated with HG (30 mM D‐glucose) and LPS (100 ng/mL; Sigma‐Aldrich, SMB00704) (co‐treatment protocol). Specifically, RAW 264.7 cells were seeded and allowed to adhere overnight. The culture medium was then replaced with fresh medium containing HG (30 mM), LPS (100 ng/mL), and PNS at the indicated concentrations (30, 100, or 200 μg/mL), and the cells were incubated for 24 h before analysis. For the mechanistic studies, TPPU (10 μM) or 14,15‐EEZE (10 μM) was also added concurrently with PNS and the HG + LPS stimuli. All in vitro treatments (HG, LPS, PNS, TPPU, and 14,15‐EEZE) were applied simultaneously for a continuous 24‐h incubation period. Cell viability was assessed by CCK‐8 assay after 24 h of treatment, and all downstream analyses were performed at the 24‐h endpoint.

### 2.10 Annexin V‐FITC/Propidium Iodide (PI) Staining

The percentage of pyroptotic cells was determined using an Annexin V‐FITC/PI Apoptosis Detection Kit (Beyotime, C1062). Additionally, this assay identifies late apoptotic and pyroptotic cells. RAW 264.7 cells (1 × 10^6^ cells per sample) were collected, washed with PBS, and resuspended in 100 μL binding buffer. The cells were incubated with 5 μL Annexin V and 10 μL PI for 15 min at room temperature in the dark. Subsequently, 400 μL binding buffer was added to each tube for immediate analysis using a Beckman flow cytometer. Pyroptotic cells were quantified as double‐positive (Annexin V^+^/PI^+^) populations using FlowJo (10.8.1) software.

### 2.11 Detection of mtROS

Intracellular and mitochondrial ROS levels in RAW264.7 cells (1 × 10^6^ cells/sample) were assessed using the fluorescent probes 5 μM MitoSOX (Med Chem Express, HY‐D1055), respectively. Samples were immediately analyzed using a Beckman flow cytometer, and the average fluorescence intensity was quantified using FlowJo software (version 10.8.1).

### 2.12 Assessment of Mitochondrial Membrane Potential (MMP)

MMP were assessed using MitoTracker Green FM (50 nM; Beyotime, C1048) and MitoTracker Red (100 nM; Beyotime, C1035), respectively. Cells (1 × 10^6^ cells/sample) were immediately analyzed using a Beckman flow cytometer after incubation with two fluorescent probes. The ratio of MitoTracker Red to Green fluorescence intensity indicated MMP.

For qualitative imaging, cells grown on chamber slides were stained with 100 nM MitoTracker Red at 37°C for 20 min and counterstained with DAPI (1 μg/mL) for 5 min to label the nuclei. Images were captured using a confocal microscope with a 63×/1.40 NA oil immersion objective.

### 2.13 Identification of the Compounds in PNS by UPLC‐QE‐Orbitrap‐MS

For sample preparation, 3 mg of PNS was dissolved in 300 μL of methanol and filtered through a 0.22 μm ultrafiltration membrane. Chromatographic separation was performed on an ACQUITY UPLC HSS T3 column (100 mm × 2.1 mm, 1.8 μm) maintained at 45°C with a flow rate of 0.35 mL/min. The mobile phase comprised 0.1% formic acid in water (solvent A) and acetonitrile (solvent B). An injection volume of 2 μL was used. The gradient elution program was applied as follows: 0–3 min, 100% A; 18.5 min, 80% A; 20 min, 65% A; 26 min, 60% A; 35 min, 5% A; 38 min, 5% A; 38.1 min, 100%; 40 min, 100% A. Mass spectrometric detection was conducted using a HESI ion source in both positive and negative ionization modes with a full‐scan range of 100–1500 *m/z* on a UPLC‐Q‐Exactive Orbitrap system. Data processing, including baseline filtering, peak detection, integration, and retention time alignment, was performed using XCMS (version 4.5.1). System suitability was monitored using pooled QC samples (prepared by mixing equal aliquots of all samples) injected at the beginning, every six samples, and at the end of each analytical batch, with retention‐time drift and signal‐intensity RSD of representative features all below 15%. The identity of each compound was established by orthogonal criteria‐retention time matching, accurate mass determination (mass error < 5 ppm), characteristic MS/MS fragmentation patterns, and database matching (HMDB, METLIN, and in‐house standard comparison).

### 2.14 Molecular Docking

The compounds' SDF files were retrieved from the PubChem database (https://pubchem.ncbi.nlm.nih.gov/). The PDB file of EPHX2 protein was downloaded from the Protein Data Bank (https://www.rcsb.org/). Molecular docking was performed using AutoDock Vina (version 1.1.2) to generate binding energies, and the results were visualized with PyMOL (version 2.3).

### 2.15 Statistical Analysis

All experimental specimens, including murine and cellular samples, were validated with a minimum of three biological replicates. Values were calculated as mean ± standard error of the mean. Statistical analyses were performed using GraphPad Prism software (version 9.5). Prior to parametric analyses, normality was assessed using the Shapiro–Wilk test and homogeneity of variances was verified using Levene's test. Comparisons between two groups were performed using unpaired Student's *t*‐test. For multi‐group comparisons (including the six‐arm experiments in Figures 4, 5, 6), one‐way analysis of variance (one‐way ANOVA) was performed, followed by Tukey's honestly significant difference (Tukey's HSD) post hoc test for pairwise multiple comparisons. A *p* < 0.05 indicated statistical significance.

## 3 Results

### 3.1 PNS Promoted Wound Healing in Diabetic Mice

In DW mice, PNS treatment exerted no significant effect on body weight or RBG levels. (Figure 1A,B). However, PNS administration significantly improved glucose tolerance and insulin sensitivity, as evidenced by GTT and ITT, including their corresponding area under the curve analyses (Figure 1C–F). Wound healing was significantly impaired in the DW group compared with the NW controls. PNS treatment markedly accelerated wound closure in a dose‐dependent manner (Figure 1G–I). Histological examination revealed that DW mice exhibited a larger epithelial gap, higher inflammatory cell infiltration, and less collagen deposition than NW mice. However, PNS treatment effectively reversed these pathological changes (Figure 1J–N). These findings indicate that PNS facilitates wound repair in diabetic mice.

**FIGURE 1:**
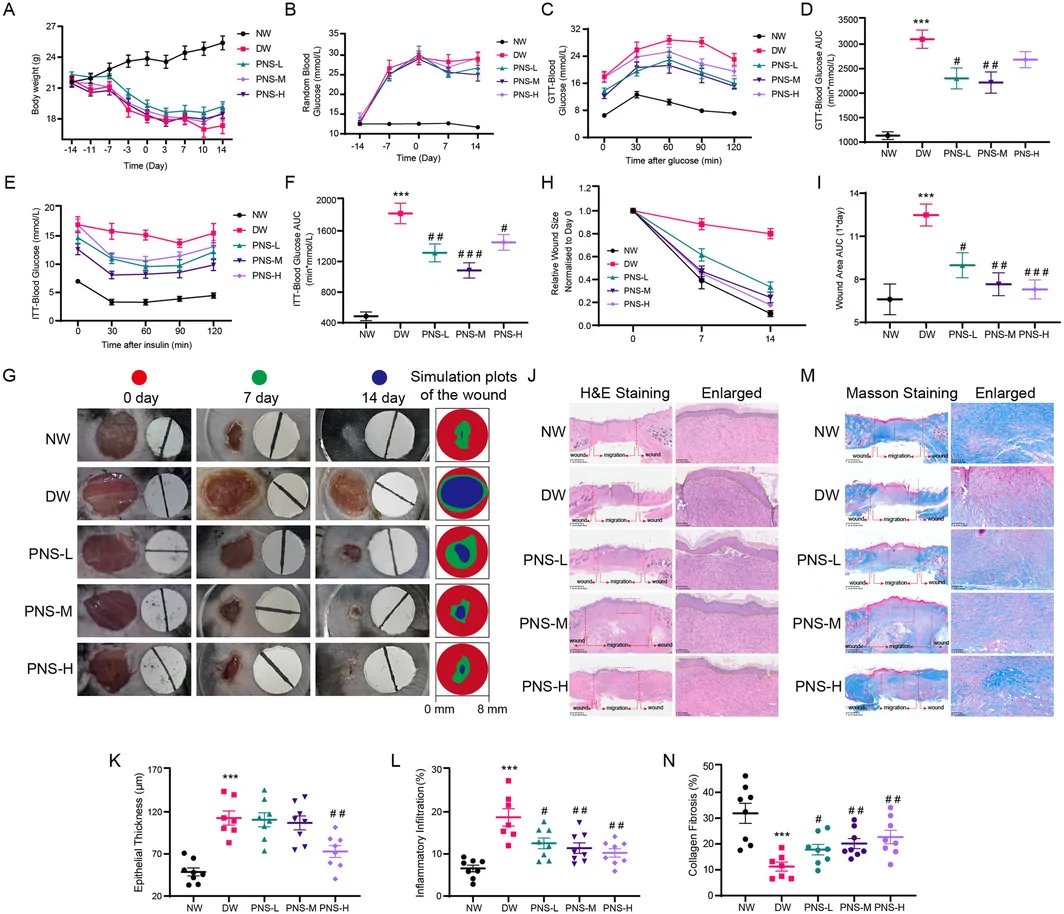
PNS promotes wound healing in DW mice. PNS treatment was initiated on day 0. STZ injection occurred on day −14, and wound establishment on day −7. (A) Body weight. (B) Random blood glucose. (C, D) GTT and corresponding AUC quantitative analysis. (E, F) ITT and corresponding AUC quantitative analysis. (G) Wound healing progression, dynamic changes in wound size. Disc diameter: 6 mm. (H, I) Wound area change and AUC quantitative analysis. (J) Representative images of H&E‐stained wound sections. Red dashed lines demarcate the wound and migration areas; the yellow dashed area outlines the epithelial thickness. Scale bars: 500 μm (left), 100 μm (right). (K, L) Quantitative analysis of epithelial thickness, and inflammatory infiltration. (M) Representative images of Masson‐stained wound sections. Red dashed lines demarcate the wound and migration areas. Scale bars: 500 μm (left), 100 μm (right) (N) Quantitative analysis of collagen deposition area. Values were expressed as means ± SEM, *n* = 6–8. ***p < 0.001, vs. NW; ^#^ *p <* 0.05, ^##^ *p <* 0.01, ^###^ *p <* 0.001 vs. DW.

### 3.2 PNS Minimized Mitochondrial Dysfunction and Inhibited Pyroptosis in Diabetic Wounds

Compared with NW mice, DW mice exhibited significantly higher concentrations of the pro‐inflammatory cytokines IL‐1β, IL‐6, and IL‐18, as well as a lower level of IL‐10. Conversely, PNS treatment significantly reduced pro‐inflammatory cytokine levels and increased IL‐10 production (Figure 2A–D). Consistent with these findings, immunofluorescence staining revealed a higher abundance of M1 macrophages and a lower abundance of M2 macrophages in wound tissues from DW mice compared with NW controls. In contrast, PNS treatment markedly reduced M1 macrophage infiltration and increased the M2 macrophage population (Figure 2E,F). Western blotting of wound tissues indicated higher iNOS and lower Arg1 expression in the DW group than in the NW group. After PNS treatment, iNOS and Arg1 expressions were significantly downregulated and upregulated, respectively (Figure 2G,H). We investigated PNS effect on NLRP3 inflammasome‐mediated pyroptosis, which is crucial to DFU pathogenesis and macrophage polarization (Llena‐Meler et al. 2026). Western blotting revealed a significant upregulation of NLRP3, pro‐Caspase‐1, the active Caspase‐1 p20 fragment, FL‐GSDMD and N‐GSDMD in wound tissues from the DW group compared to those from the NW group. Besides, PNS treatment significantly reduced the protein levels of these key pyroptosis‐related markers (Figure 2I–K). Furthermore, immunohistochemical measurement for GSDMD in wound migration further corroborated these findings (Figure 2L,M). Mitochondria are the primary site of cellular respiration (Kisty et al. 2023). In a hyperglycemic environment, mitochondria promote ROS accumulation and lead to mitochondrial dysfunction, while pyroptosis typically originates from mitochondrial dysfunction (Kaur et al. 2026; Weindel et al. 2022). Transmission electron microscopy (TEM) of wound tissue revealed severe ultrastructural damage characterized by nuclear membrane fragmentation, electron‐dense bodies, and swollen mitochondria, which significantly improved after PNS treatment (Figure S2). At the molecular level, the mitochondrial dynamics balance was disrupted in DW mice, as evidenced by a significantly increased p‐Drp1 (Ser616): total Drp1 ratio (indicating enhanced fission) and decreased protein expression of the fusion mediators Mfn1, Mfn2, and OPA1. Treatment with PNS effectively restored the balance, reduced Drp1 activation, and encouraged mitochondrial fusion (Figure 2N,O). Collectively, the TEM ultrastructural analysis, and expression profiling of mitochondrial dynamics proteins (Drp1, Mfn1, Mfn2, OPA1) demonstrate that PNS minimizes not only functional but also structural mitochondrial dysfunction in diabetic wounds.

**FIGURE 2:**
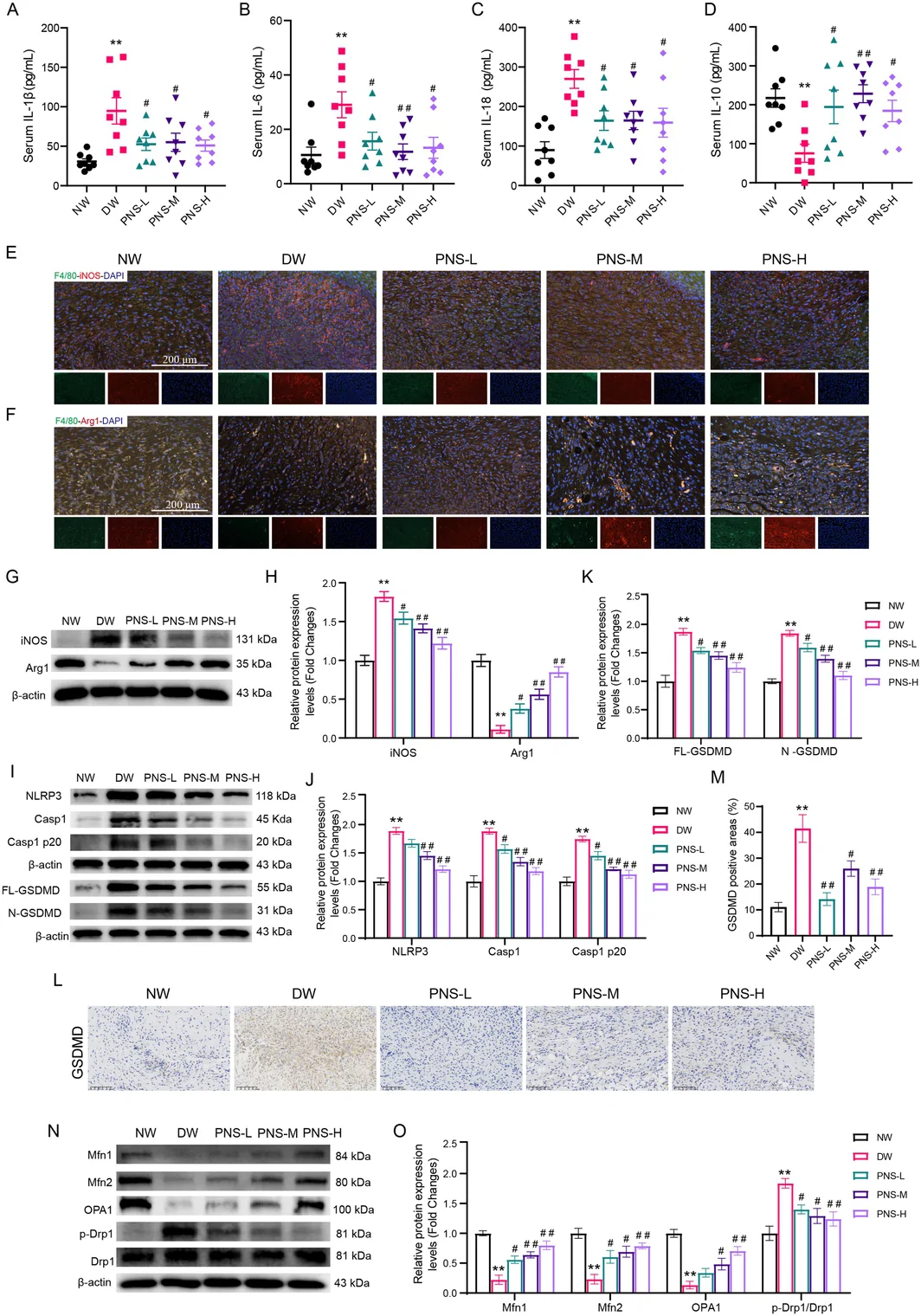
PNS inhibits pyroptosis and mitochondrial homeostasis in wound tissue of DW mice. (A–D) ELISA was used to measure serum levels of IL‐1β, IL‐6, IL‐18, and IL‐10. (E) Immunofluorescence staining of wound sections identified M1 macrophages using antibodies against F4/80 and iNOS. Scale bar: 200 μm. (F) Immunofluorescence staining of wound sections identified M2 macrophages using antibodies against F4/80 and Arg1. Scale bar: 200 μm. (G, H) Western blotting detected protein expression of iNOS and Arg1 in wound tissues, followed by quantitative analysis. (I–K) Western blotting detected protein expression of NLRP3, Casp1, Casp1 p20, FL‐GSDMD, and N‐GSDMD in wound tissues, followed by quantitative analysis. (L, M) Immunohistochemical staining and quantitative analysis of GSDMD expression in wound sections. (N, O) Western blotting detected protein expression of Mfn1, Mfn2, OPA1, p‐Drp1, and Drp1 in wound tissues, followed by quantitative analysis. Values were expressed as means ± SEM, *n* = 6–8. **p < 0.01, vs. NW; ^#^ *p <* 0.05, ^##^ *p <* 0.01 vs. DW.

### 3.3 PNS Increased 11,12‐EET Levels by Inhibiting Ephx2

To uncover the metabolic alterations underlying the therapeutic effect of PNS, we performed untargeted metabolomics on wound tissues. Volcano plot analysis identified 67 significantly dysregulated metabolites in DW tissues compared with NW tissues (39 upregulated and 28 downregulated). PNS treatment restored the abundance of 17 of these metabolites (10 upregulated and 7 downregulated) to normal levels (Figure 3A,B). Notably, Venn diagram analysis identified four metabolites that were significantly altered in DW versus NW and PNS versus DW comparisons. These metabolites are considered key nodes in PNS intervention, particularly 11,12‐EET (Figure 3C–E), which has been identified as crucial for wound healing (Chi et al. 2024; Zhao et al. 2017). In addition, we further found a trend of changes in 11,12‐DHET before and after PNS treatment, and 11,12‐EET/11,12‐DHET showed a significant pivotal role, suggesting that the degradation of 11,12‐EET may play an important role in PNS (Figure S3).

**FIGURE 3:**
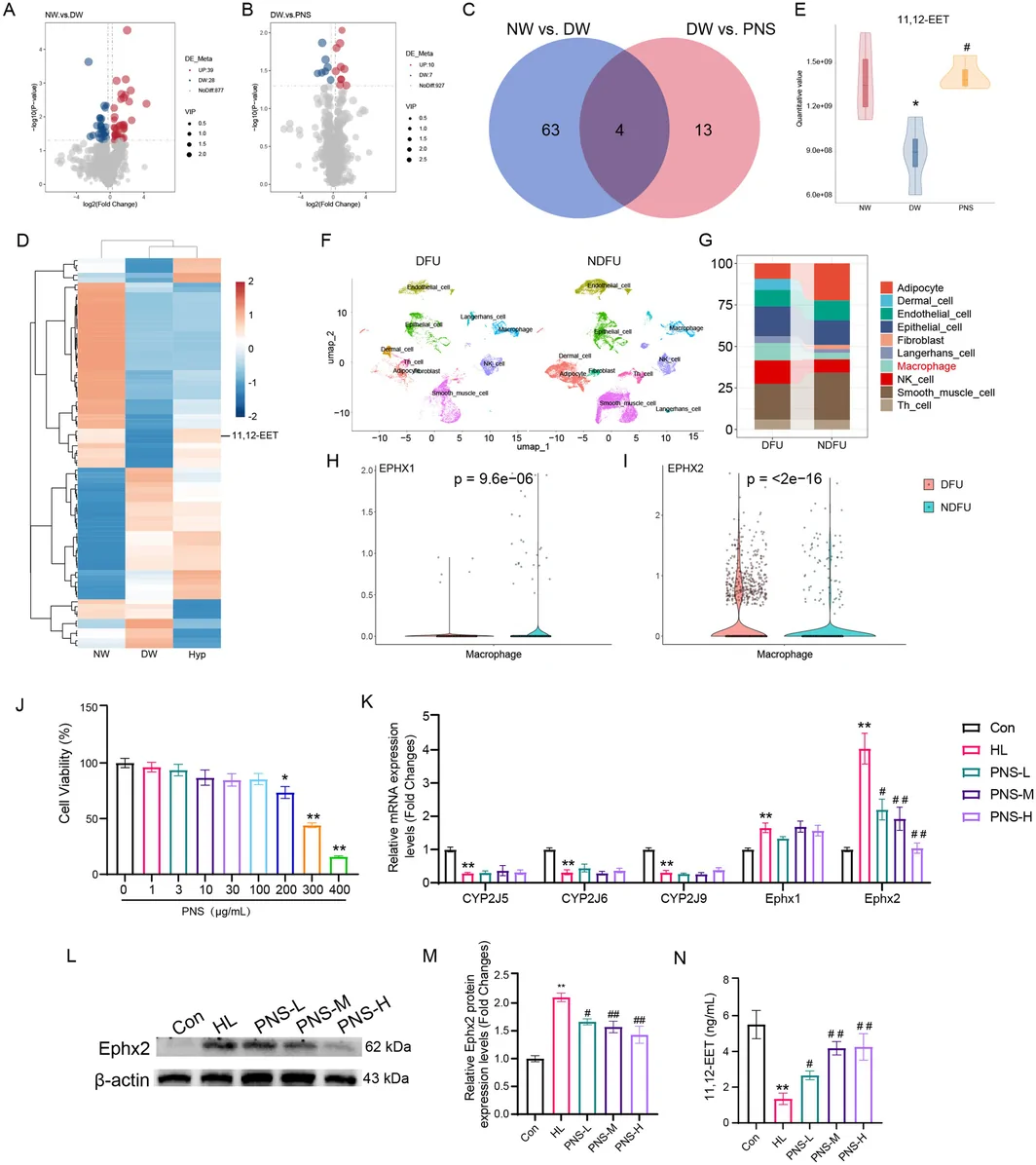
Ephx2/11,12‐EET is a key metabolic axis in the PNS promoting wound healing in DW mice. (A, B) Volcano plot analysis of differential metabolites between the NW and DW groups, and between the DW and PNS groups. (C) Venn diagram identifying common differential metabolites between the NW vs. DW comparison and the DW vs. PNS comparison. (D) Heatmap displaying the common differential metabolites. (E) Quantitative values of 11,12‐EET. (F) UMAP plots present the cell clusters in foot skin tissues from the DFU and NDFU cohorts in GSE165816 dataset. (G) Bar plots compare the proportional abundance of these cell clusters between the DFU and NDFU groups. (H, I) Differential expression of EPHX1 and EPHX2 in macrophages from the NDFU and DFU groups in GSE165816 dataset. (J) Cell viability of RAW264.7 cells was measured using the CCK‐8 assay. (K) mRNA expression levels of Cyp2j5, Cyp2j6, Cyp2j9, Ephx1, and Ephx2 in RAW264.7 cells were detected via qPCR. (L, M) Western blotting detected protein expression of Ephx2 in RAW264.7 cells with PNS‐L (30 μg/mL), PNS‐M (100 μg/mL), or PNS‐H (200 μg/mL), followed by quantitative analysis. (N) ELISA was used to determine the level of 11,12‐EET in cell culture supernatant. Values were expressed as means ± SEM, *n* = 4–6. *p < 0.05, **p < 0.01, vs. NW; ^#^ *p* < 0.05, ^##^ *p* < 0.05, vs. DW.

11,12‐EET is a metabolite produced by the metabolism of arachidonic acid via CYP450 epoxidases, particularly the Cyp2J family. Simultaneously, it is degraded by sEH, primarily Ephx1 and Ephx2. We selected foot skin samples from diabetic patients with non‐healing wounds (DFU) and healthy subjects (NDFU) from the public scRNA dataset (GSE165816). UMAP successfully identified major cell populations, including macrophages, fibroblasts, and other cells (Figure 3F,G). Unfortunately, no genes from the Cyp2J family were detected in the GSE165816. However, we observed a pronounced decrease in Ephx1 expression and a noticeable increase in Ephx2 expression in the DFU group compared to the NDFU group (Figure 3H,I). We confirmed that 100 μg/mL PNS did not inhibit the growth of RAW 264.7 cells in an in vitro model stimulated by high glucose and lipopolysaccharide (HL) (Figure 3J). Additionally, qPCR analysis revealed that PNS treatment selectively reversed Ephx2 gene expression, rather than Ephx1 gene expression or the Cyp2J family (Figure 3K). Furthermore, we confirmed that PNS regulates Ephx2 protein expression in macrophages challenged with HL. Additionally, PNS enhances the thermal stability of Ephx2 protein in macrophages, suggesting the possibility of an intracellular interaction between Ephx2 and PNS (Figure 3L,M; Figure S4). In addition, HL stimulation significantly reduced 11,12‐EET level in the cell culture supernatant and was reversed by PNS treatment in a concentration‐dependent manner (Figure 3N).

### 3.4 PNS Suppressed Pyroptosis and Minimized Mitochondrial Dysfunction of Macrophage via Regulating Ephx2/11,12‐EET Axis

Based on these findings, we selected TPPU, an Ephx2 inhibitor, for testing. Notably, both PNS and TPPU significantly enhanced the effects of 11,12‐EET. Furthermore, TPPU only marginally amplified the pharmacological effects of PNS, without statistically significant difference (Figure 4A). Macrophages clearly exhibit morphological activation under HL challenge (Figure 4B), confirmed as M1‐type activation primarily due to high CD86 expression (Figure 4C), which is reversed by PNS and TPPU. Concomitantly, PNS and TPPU suppressed the secretion of pro‐inflammatory cytokines (IL‐1β, IL‐6, IL‐18) (Figure 4E–G) and enhanced the secretion of anti‐inflammatory cytokines (IL‐10) (Figure 4H) in macrophages challenged with HL. Notably, TPPU did not significantly enhance the pharmacological effects of PNS, suggesting that PNS and TPPU may share common targets. Annexin V/PI staining revealed that HL stimulation significantly increased the percentage of pyroptotic cells. This was markedly reduced by PNS treatment. TPPU produces a similar effect but does not significantly enhance the action of PNS (Figure 4I,J). At the molecular level, these pyroptotic markers, including NLRP3, Caspase‐1, Casp1 p20, FL‐GSDMD, and N‐GSDMD, were effectively suppressed by PNS treatment, which was not further enhanced by co‐treatment with TPPU (Figure 4K–M). More intuitively, Immunofluorescence experiments also confirmed that GSDMD suppressed by PNS in HL‐stimulated macrophages was not enhanced by TPPU (Figure 4N,O). Investigations into the upstream mechanisms of pyroptosis yielded consistent results: PNS treatment restored the equilibrium state of mitochondrial dynamic proteins, including promoting mitochondrial fusion (Mfn1, Mfn2, and OPA1) and inhibiting mitochondrial fission (Drp1). This effect was not further enhanced by TPPU treatment (Figure 4P,Q). This further suggests that Ephx2 mediates PNS‐induced 11,12‐EET production and is critical for its pharmacological effects.

**FIGURE 4:**
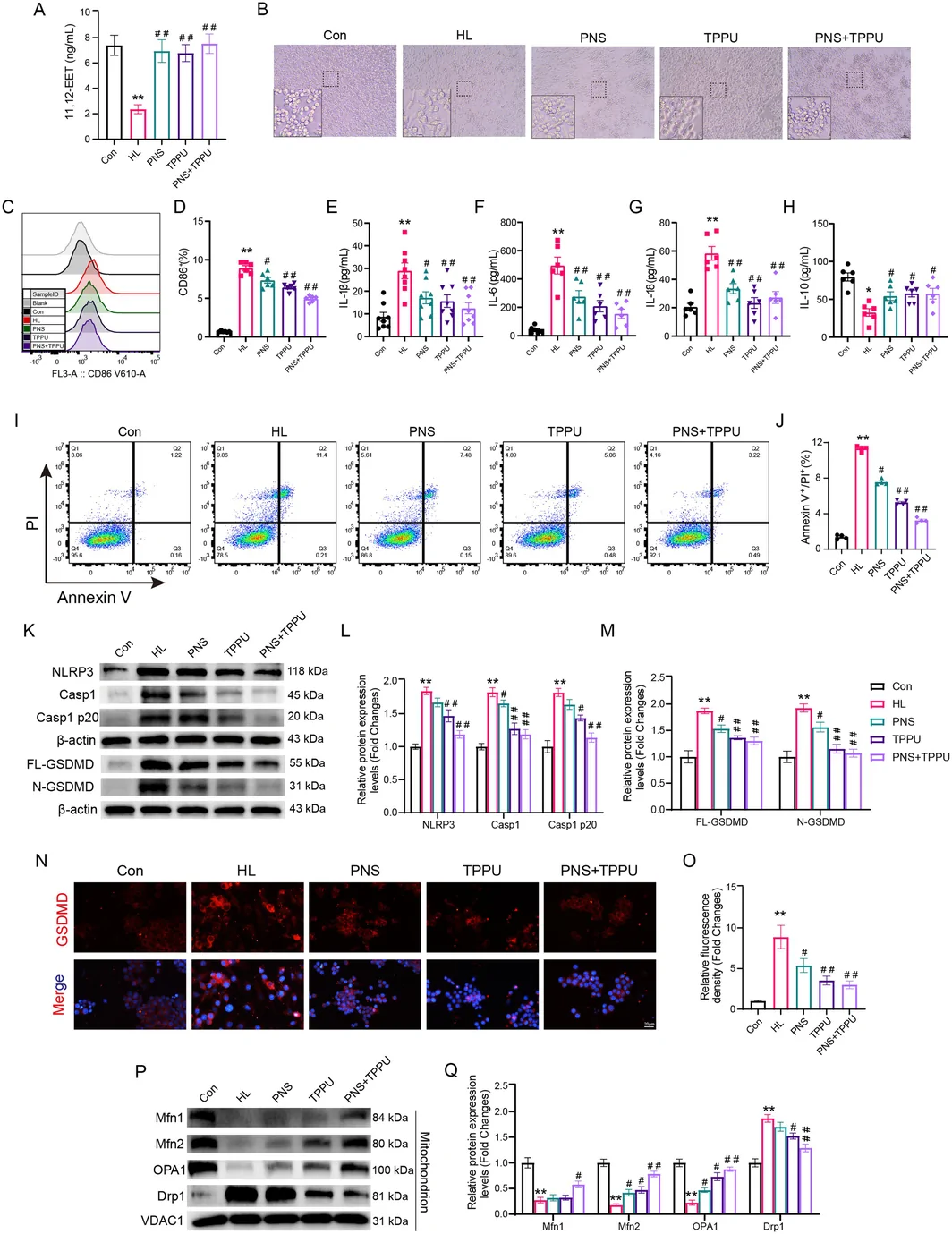
TPPU reduces macrophage pyroptosis and mitochondrial homeostasis without enhancing the efficacy of PNS. (A) ELISA was used to determine the level of 11,12‐EET in cell culture supernatant. (B) Representative images show macrophage differentiation. (C, D) CD86 levels in macrophages were analyzed by flow cytometry, accompanied by quantitative analysis. (E–H) ELISA was used to determine the level of IL‐1β, IL‐6, IL‐18, and IL‐10 in cell supernatant. (I, J) Flow cytometry was used to detect Annexin V/PI double‐positive regions in RAW264.7 cells, followed by quantitative analysis. (K–M) Western blotting was performed to assess the intracellular expression levels of NLRP3, Casp1, Casp1 p20, FL‐GSDMD, and N‐GSDMD, followed by quantitative analysis. (N, O) Immunofluorescence staining was carried out to detect GSDMD expression in RAW264.7 cells, followed by quantitative analysis. Scale bar: 20 μm. (P, Q) Western blotting was performed to assess the intracellular expression levels of Mfn1, Mfn2, OPA1, p‐Drp1, and Drp1, followed by quantitative analysis. Values were expressed as means ± SEM, *n* = 4–6. **p < 0.01, vs. Con; ^#^ *p <* 0.05, ^##^ *p <* 0.01 vs. HL.

### 3.5 Loss of 11,12‐EET Function Blocked the PNS's Protective Effects on Mitochondria

We further employed the 11,12‐EET inhibitor 14,15‐EEZE to block PNS regulation of 11,12‐EET in macrophages (Figure 5A). Notably, 14,15‐EEZE completely reversed PNS‐mediated suppression of mtROS (Figure 5B,C), along with its protective effects on mitochondrial morphology, manifested by a reduction in the relative length of mitochondria (Figure 5D, Figure S5A). Additionally, we found that the PNS‐induced upregulation of mitochondrial fusion genes (Mfn1, Mfn2, and OPA1) and the concomitant downregulation of the fission gene Drp1 in macrophages under HL challenge were reversed by 14,15‐EEZE (Figure S5B). Furthermore, OCR and ECAR measurements confirmed that the PNS‐induced enhancement of mitochondrial function was blocked by 14,15‐EEZE but not significantly enhanced by TPPU (Figure 5D). Additionally, 14,15‐EEZE abolished the PNS‐mediated regulation of mitochondrial membrane potential in HL‐stimulated macrophages, and TPPU still failed to enhance the PNS pharmacological effects. This further indicates that the Ephx2/11,12‐EET axis is a crucial pathway for the PNS to exert its pharmacological effects (Figure 5G,H).

**FIGURE 5:**
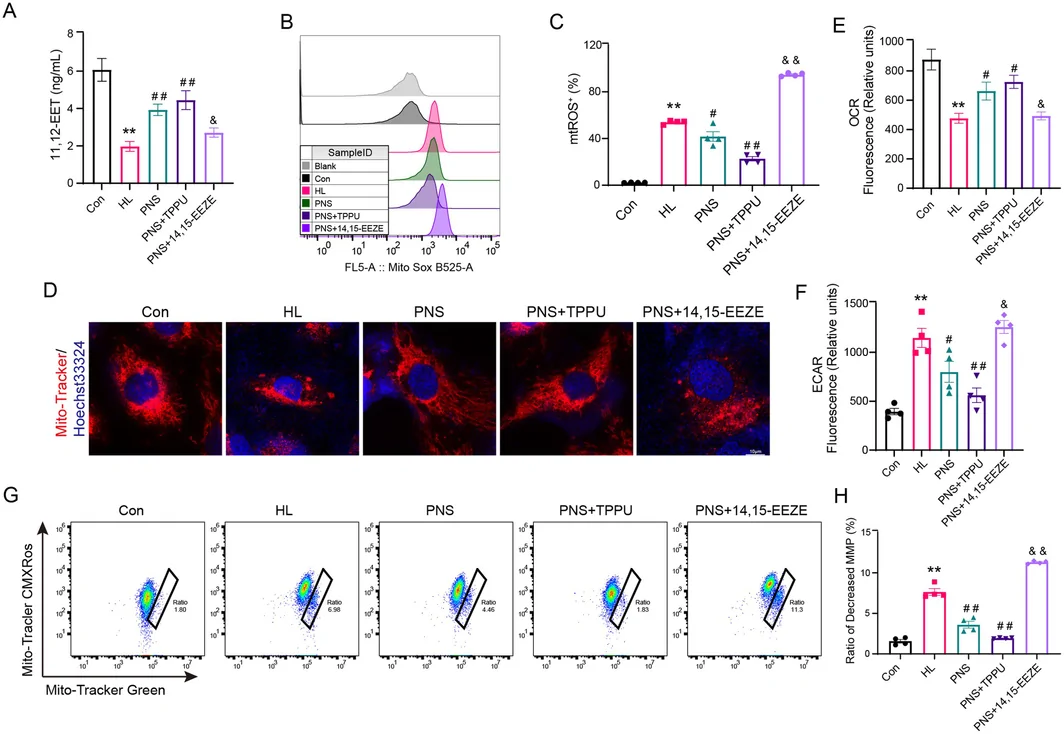
14,15‐EEZE weakens the protection of the PNS against mitochondrial homeostasis. (A) ELISA was used to measure the level of 11,12‐EET in the supernatant of RAW264.7 cells. (B, C) The detection of mitoSOX‐positive areas reflects the mtROS levels in RAW264.7 cells, followed by quantitative analysis. (D) Mitochondrial status was visualized using MitoTracker staining and immunofluorescence in RAW264.7 cells. (E, F) The OCR and ECAR in RAW264.7 cells was measured using ELISA. (G, H) Fluorescence in the MitoTracker CMXRos/MitoTracker Green assay was assessed by flow cytometry, accompanied by quantitative analysis. Values were expressed as means ± SEM, *n* = 4–6. **p < 0.01, vs. Con; ^#^ *p <* 0.05, ^##^ *p <* 0.01 vs. HL; ^&^ *p <* 0.05, ^&&^ *p* < 0.05, vs. PNS.

### 3.6 11,12‐EET Emerged as the Key Mediator for PNS Efficacy In Vivo Experiments

To validate the pathological relevance of our in vitro findings, we assessed the role of 11,12‐EET in the therapeutic effects of PNS in vivo. Co‐administration of TPPU with PNS did not significantly enhance wound closure in DW mice compared with PNS treatment. Conversely, 14,15‐EEZE completely prevented PNS' pro‐healing effect in DW mice (Figure 6A–C). Additionally, PNS significantly elevated 11,12‐EET protein levels while suppressing Ephx2 mRNA expression in wound tissue of DW mice. However, these pharmacological effects were abolished by 14,15‐EEZE but not further potentiated by TPPU (Figure 6D,E). Masson staining revealed that the improved collagen organization and density induced by PNS were not amplified by TPPU but were substantially disrupted by 14,15‐EEZE (Figure 6F,G). Furthermore, immunohistochemical analysis demonstrated that the suppression of GSDMD expression by PNS was not potentiated by TPPU and reversed by 14,15‐EEZE (Figure 6H,I). These in vivo gain‐of‐function and loss‐of‐function experiments prove that 11,12‐EET plays a crucial role in the therapeutic effects of PNS on diabetic wound healing.

**FIGURE 6:**
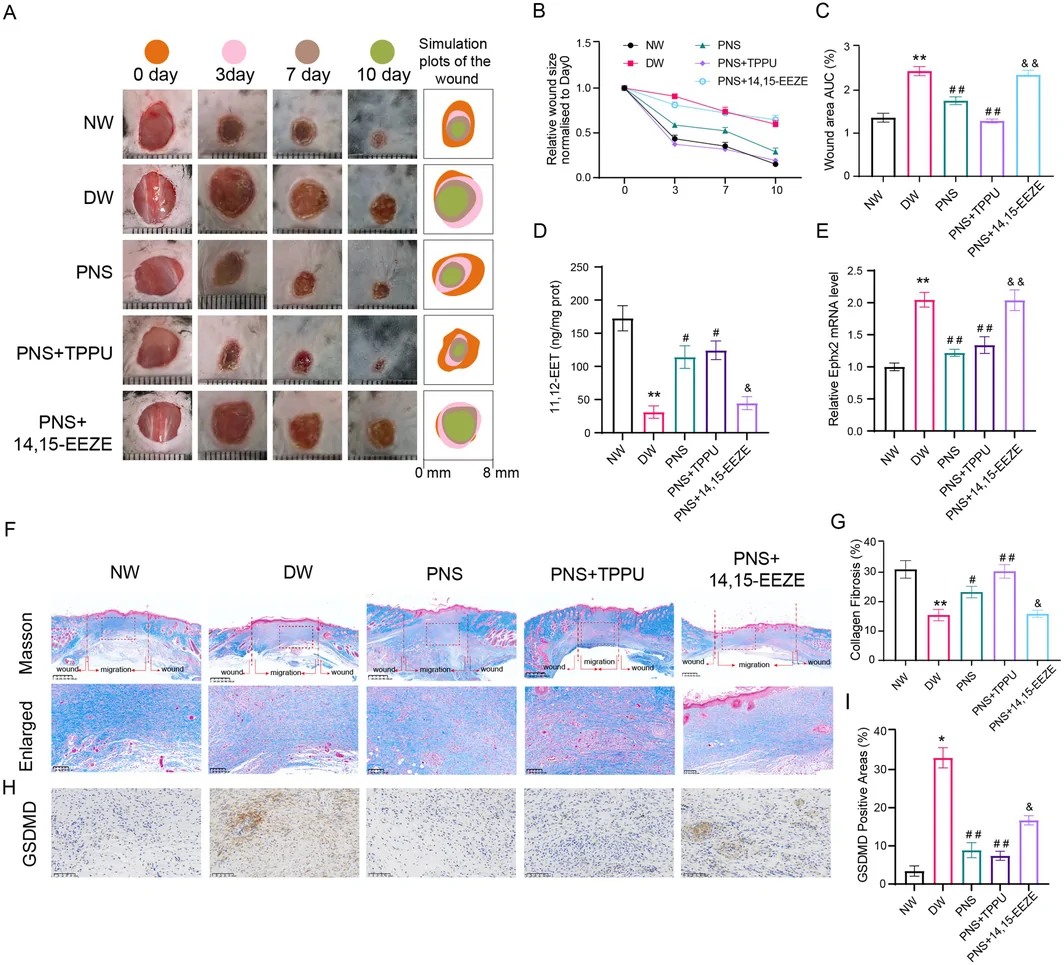
11,12‐EET mediates the therapeutic effects of PNS on diabetic wound healing. (A) Schematic illustration of dynamic wound healing progression across different stages. (B, C) Quantitative analysis of wound closure and corresponding AUC calculation. (D) qPCR detection of Ephx2 gene expression levels in wounds. (E) ELISA detection of 11,12‐EET levels in wound tissue. (F, G) Masson staining of wound tissues with quantitative evaluation of collagen deposition. Red dashed lines demarcate the wound and migration areas. Scale bar: 500 μm (upper), 100 μm (lower). (H, I) Immunohistochemical detection of GSDMD expression in wound tissues and quantitative analysis. Scale bar: 100 μm. Values were expressed as means ± SEM, *n* = 6–8. **p < 0.01, vs. NW; ^#^ *p <* 0.05, ^##^ *p <* 0.01 vs. DW; ^&^ *p <* 0.05 vs. PNS.

### 3.7 Identifying Active Saponin Components Targeting the Ephx2/11,12‐EET Axis in PNS

To identify the specific constituents responsible for PNS efficacy, we performed UPLC‐QE‐Orbitrap‐MS analysis. Chromatographic profiling and quantification revealed five relatively abundant saponins in the PNS extract, including R1 (15.90%), Rg1 (3.80%), Re (3.85%), (4.07%), and Rd. (2.55%) (Figure 7A,B). Components of UPLC‐Q‐TOFMS analysis are listed in Table S3. Molecular docking simulations predicted that all five saponins could strongly bind to Ephx2, with high binding affinities (binding energies: −9.0 to −8.5 kcal/mol), suggesting their potential to inhibit sEH and regulate 11,12‐EET levels (Figure S6A). In addition, we also found that all saponins have the effect of inhibiting Ephx2 mRNA (Figure S6B) production, reducing the production of 11,12‐EET (Figure S6C).

**FIGURE 7:**
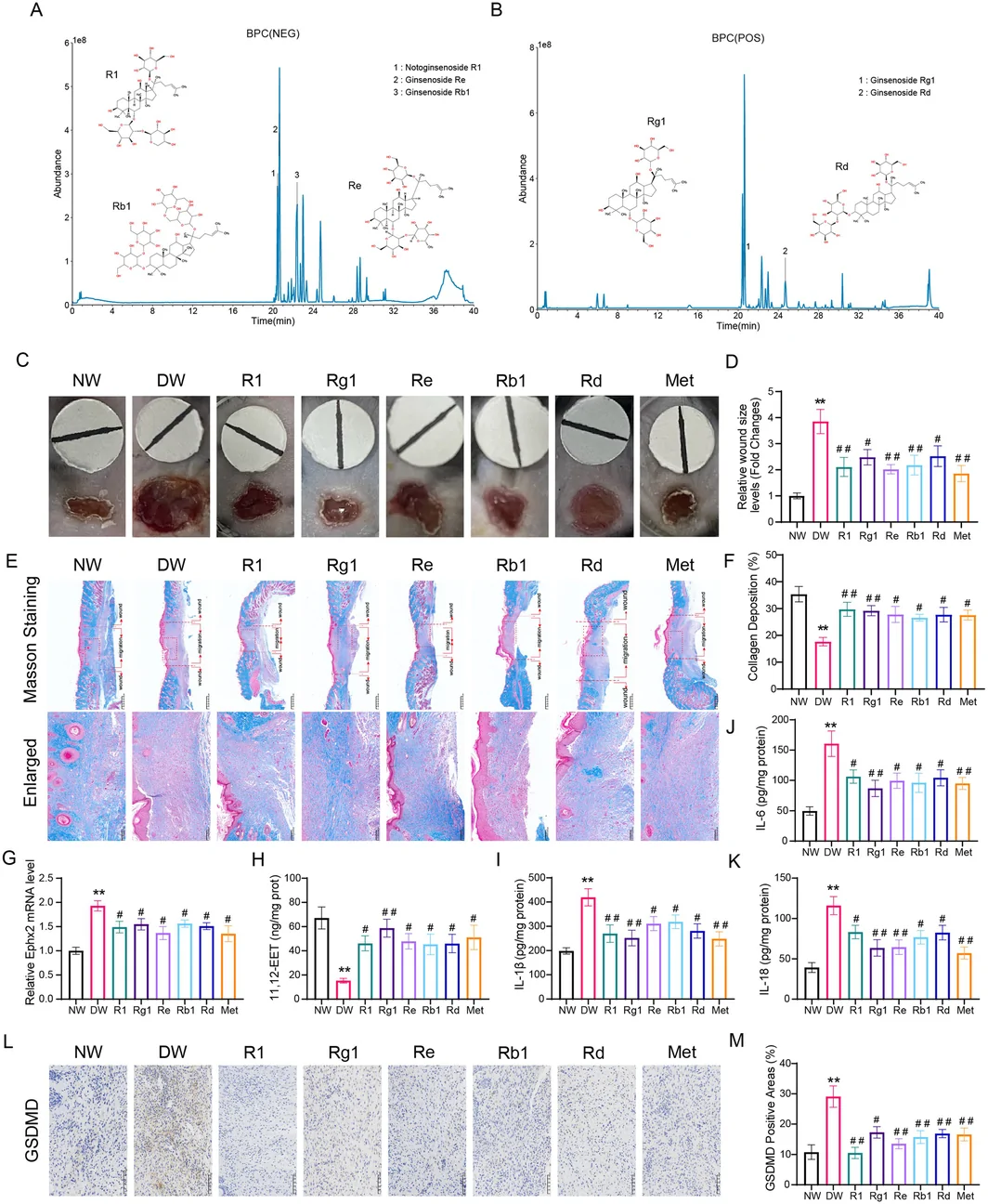
Identification of active PNS constituents that promote wound healing. (A, B) Representative base peak chromatograms (BPC) in positive and negative ion modes are shown, with the top five compounds by peak area labeled. (C, D) Representative images of wounds in DW mice and quantitative analysis of wound area. (E, F) Collagen deposition in wound tissues was assessed using Masson staining and quantified. Red lines demarcate the wound and migration areas. Scale bar: 500 μm (upper), 100 μm (lower). (G) qPCR analysis of Ephx2 mRNA in the wound tissues. (H–K) ELISA was used to measure protein levels of 11,12‐EET, IL‐1β, IL‐6, and IL‐18 in the wound tissues. (L, M) Immunohistochemical detection of GSDMD expression in wound tissues and quantitative analysis. Scale bar: 100 μm. Values were expressed as means ± SEM, *n* = 6–8. **p < 0.01, vs. NW; ^#^ *p* < 0.05, ^##^ *p* < 0.01 vs. DW.

We evaluated the individual therapeutic effects of these five constituents in the DW mouse model. Administration of individual saponin (at 10 mg/kg) markedly accelerated wound closure compared with the vehicle‐treated DW group. The observed efficacy corresponds with that of the positive control metformin (200 mg/kg) (Figure 7C,D). Similarly, Masson staining demonstrated that all five compounds significantly facilitated collagen deposition in the wound beds of DW mice (Figure 7E,F). Equally, we found that these five saponins inhibit Ephx2 mRNA expression (Figure 7G) while promoting 11,12‐EET expression (Figure 7H) in wound sites of DW mice. Treatment with five saponins significantly reduced the levels of IL‐1β (Figure 7I), IL‐6 (Figure 7J), and IL‐18 (Figure 7K) in the wounds of DW mice, as well as the positive expression of GSDMD (Figure 7L,M). This further indicates that the five saponins form a crucial foundation for the therapeutic effects of PNS.

## 4 Discussion

Diabetic wound healing impairment represents one of the major clinical challenges faced by individuals with diabetes (McDermott et al. 2023), with its pathological mechanisms involving multiple factors such as chronic inflammation, metabolic disorders, and cellular dysfunction (Clayton et al. 2024). This study systematically investigated the mechanisms by which PNS promotes diabetic wound healing, revealing that PNS improves mitochondrial homeostasis and inhibits pyroptosis by regulating the Ephx2/11,12‐EET axis in macrophages.

The healing process of diabetic wounds primarily encompasses the inflammatory, proliferative, and maturation phase (Britto et al. 2026). However, the initiation and progression of this process are profoundly influenced by the inflammatory response, with macrophages playing an absolutely central role (Audu et al. 2022). M1 activation of macrophages represents the culmination of pyroptosis, characterized by mitochondrial dysfunction and abnormal NLRP3 inflammasome activation. This process triggers the release of pro‐inflammatory cytokines such as IL‐1β and IL‐18, which are crucial for recruiting additional immune cells and exacerbating local inflammatory responses (Islamuddin and Qin 2024). Noteworthily, PNS reduces abnormal weight loss in diabetic mice and improves insulin sensitivity, even without significantly lowering random blood glucose levels. This is crucial for diabetic mice, as the relative integrity of insulin function is a critical strategy for reducing other complications such as diabetic nephropathy and impaired wound healing (Allison 2017; D'Agostino et al. 2025). Noticeably, we demonstrated in diabetic mice models that PNS significantly accelerates wound closure and improves histological structure, including reduced inflammatory cell infiltration and increased collagen deposition. Current clinical applications of PNS primarily leverage its anti‐inflammatory and antioxidant properties (Li et al. 2024).

However, current research on the therapeutic role of PNS in promoting diabetic wound healing has primarily focused on angiogenesis and ECM remodeling rather than macrophage activity (Lei et al. 2022), which constitutes the novelty of this study. Mechanistically, we found that PNS reduces the accumulation of M1 macrophages in diabetic wounds, along with the secretion of pro‐inflammatory factors and pyroptosis. Multiple studies have confirmed that PNS can downregulate the expression of several key proteins in the NLRP3 inflammasome‐mediated pyroptosis pathway in a dose‐dependent manner. This effect has been demonstrated in models of cerebral ischemia–reperfusion injury, lipopolysaccharide‐induced renal tubular epithelial cell fibrosis, and hypoxia‐induced cardiomyocyte injury (Xu et al. 2023). Crucially, NLRP3 inflammasome activation is a pivotal event in the inflammatory response, with mitochondrial dysfunction and excessive ROS production serving as key signals that trigger this pathway (Dominic et al. 2022). Further in‐depth research, TEM images reveal signature ultrastructural features of pyroptosis, such as pyroptotic bodies and damaged mitochondria, while PNS reverses these features, providing intuitive and compelling support for its anti‐pyroptotic effects. Conspicuously, mitochondrial homeostasis is not only a crucial component in the progression of inflammatory responses but also an upstream regulator of NLRP3 inflammasomes (Zhang, Xiao, et al. 2025). Mitochondrial dynamics imbalance is a key driver of pyroptosis, a process primarily mediated by Drp1. When Drp1 is recruited to the outer mitochondrial membrane and activated through phosphorylation (Ser616), it causes a decline in mitochondrial membrane potential, leading to mitochondrial dysfunction (Peixoto‐Rodrigues et al. 2025). Correspondingly, impaired mitochondrial fusion prevents damaged mitochondria from being effectively integrated into functional mitochondria. Directly, damaged mitochondria generate substantial mtROS, which not only serve as damage signals but also directly promote NLRP3 inflammasome assembly (Casey et al. 2025). This ultimately drives mitochondrial membrane contraction and fragmentation. This persistent inflammatory state directly impairs the function of dermal fibroblasts and endothelial cells, which are essential for collagen deposition and angiogenesis, respectively. Specifically, elevated IL‐1β has been shown to inhibit fibroblast proliferation and migration, suppress Type I collagen synthesis, and induce fibroblast senescence and apoptosis in diabetic wound tissue (Sugioka et al. 2025). IL‐18, while less extensively studied in wound healing, has been reported to inhibits the gene expression and protein production of Type I and III collagen in human dermal fibroblasts. Furthermore, N‐GSDMD‐mediated membrane pores and the release of mtROS and oxidized mitochondrial DNA from pyroptotic macrophages can directly induce oxidative stress in adjacent fibroblasts and endothelial cells, exacerbating mitochondrial dysfunction and impairing their reparative functions (Kim et al. 2010). By inhibiting macrophage pyroptosis, PNS reduces the release of N‐GSDMD, IL‐1β, IL‐18, and DAMPs into the wound microenvironment, thereby breaking this vicious inflammatory cycle. The resulting reduction in pro‐inflammatory signaling allows dermal fibroblasts to recover their proliferative and collagen‐synthetic capacity. Simultaneously, the attenuation of oxidative and inflammatory stress on endothelial cells creates a permissive environment for VEGF‐mediated angiogenesis, which is essential for nutrient and oxygen delivery to the healing wound. The stability of structure is the premise and foundation of mitochondrial function, as well as the key to reducing downstream pyroptosis and tissue ECM repair. PNS promotes the minimization of mitochondrial structural dysfunction, accelerates the effective glucose metabolism process of mitochondria, promotes oxidative phosphorylation, and allows wounds to obtain more energy, which is the basis for wound repair (Yu et al. 2026). This may also be a key factor contributing to the restoration of mitochondrial integrity in wounds and the reduction of NLRP3‐mediated pyroptosis.

Recent studies have revealed that modulating local metabolites at the wound site can effectively improve mitochondrial function, thereby breaking the vicious cycle of healing barriers and offering new therapeutic strategies (Wu et al. 2025). Through untargeted metabolomics, we identified 11,12‐EET, an epoxy‐20‐trienoyl acid, as a potential key factor in PNS's improvement of mitochondrial function and attenuation of diabetic wound pyroptosis. 11,12‐EET has been demonstrated to be released through GSDMD‐mediated pores and participate in tissue repair processes (Chi et al. 2024). However, whether 11,12‐EET participates in GSDMD‐mediated pyroptosis and the underlying mechanisms, particularly how the PNS regulates 11,12‐EET production, remain unclear. In the diabetic wounds mouse model, 11,12‐EET levels were significantly reduced but restored post‐PNS treatment. 11,12‐EET is a key active metabolite produced by the metabolism of arachidonic acid via the CYP450 oxidase pathway, primarily involving the CYP2J subfamily. It is subsequently hydrolyzed and inactivated by soluble epoxide hydrolase. Research reveals that in diabetic (ob/ob) mouse wound granulation tissue, the expression levels of key CYP epoxides, such as CYP2J6, are significantly lower than those in normal mice. This reduction in endogenous beneficial metabolites may be associated with impaired wound healing in diabetes (Zhao et al. 2017). Additionally, Ephx2 gene knockout significantly suppressed diabetes‐induced vascular calcification by increasing levels of EETs (particularly 11,12‐EET and 14,15‐EET) in the aorta (Cai et al. 2025). However, we did not detect any genes belonging to the Cyp2J subfamily in the GSE615816 dataset. Fortunately, we observed significant differences in Ephx1 and Ephx2 expression between DFU and NDFU subjects. The RAW264.7 macrophage experiment further confirmed that PNS regulates Ephx2 transcription and translation, rather than the Cyp2J family or Ephx1. Critically, PNS enhances the thermal stability of Ephx2 protein, which is crucial for understanding the pharmacological mechanism of PNS and subsequent screening of pharmacodynamic substrates.

To determine whether the Ephx2/11,12‐EET axis is a key signaling pathway for PNS in protecting cellular mitochondrial homeostasis and preventing pyroptosis, we employed TPPU, an Ephx2 inhibitor. Notably, we confirmed the roles of PNS and TPPU in stimulating 11,12‐EET secretion in macrophages under HL stimulation. However, TPPU did not further enhance the effect of PNS on macrophage 11,12‐EET secretion, suggesting that Ephx2 may be a common target for both PNS and TPPU. Similarly, in investigating the effects of 11,12‐EET on macrophage M1 polarization (including CD86 expression and inflammatory cytokine release), we observed that PNS exerts effects comparable to TPPU. This similarity is primarily because no significant differences in efficacy were observed when the two agents were used in combination. Mechanistically, we similarly observed that HL‐induced mitochondrial dysfunction and NLRP3 inflammasome‐mediated pyroptosis in macrophages were regulated by both PNS and TPPU, without a significant synergistic enhancement. Additionally, we recruited 14,15‐EEZE to block the pharmacological effects of 11,12‐EET and observed its impact on mitochondrial homeostasis regulated by the PNS. Notably, 14,15‐EEZE blocked PNS‐mediated mitochondrial protection, including increased mitochondrial ROS production, disrupted mitochondrial energy balance, impaired mitochondrial morphology, and reduced mitochondrial membrane potential. Notably, the OCR directly reflects the rate of oxidative phosphorylation in cellular mitochondria. As a key parameter for assessing mitochondrial respiratory function, it reveals the activity of the mitochondrial respiratory chain, ATP synthesis efficiency, and overall mitochondrial health. ECAR primarily reflects the activity of cellular glycolysis, serving as a direct indicator for assessing glycolytic flux. A significant decline in OCR parameters constitutes direct evidence of impaired mitochondrial function (Marcos‐Ríos et al. 2025). In response to mitochondrial damage, cells often compensate for insufficient ATP production by upregulating glycolysis (elevated ECAR), a form of metabolic reprogramming (Oller et al. 2021). Furthermore, strong oxidative phosphorylation supports an anti‐inflammatory, M2‐polarized state via STAT6 activation, whereas a glycolytic metabolism triggers pro‐inflammatory M1 polarization via STAT1/NF‐κB (Lu et al. 2025; Ying et al. 2021). Based on our research findings, we have preliminarily demonstrated that the Ephx2/11,12‐EET axis is crucial for PNS's protection of macrophage mitochondrial function and prevention of pyroptosis.

To investigate the role of Ephx2/11,12‐EET in diabetic wound healing, we similarly employed TPPU and 14,15‐EEZE to interfere with PNS treatment in diabetic wound mice. Over a ten‐day observation period, we once again confirmed that PNS accelerated wound healing in diabetic mice. Importantly, TPPU did not significantly enhance the efficacy of PNS. Crucially, 14,15‐EEZE completely abolished the beneficial effect of PNS on wound closure in diabetic mice. Furthermore, we observed that the regulatory effect of PNS on the Ephx2/11,12‐EET axis at wound sites in diabetic mice was not enhanced by TPPU, but was completely suppressed by 14,15‐EEZE. Likewise, the wound healing effects of PNS in diabetic mice were significantly impaired by 14,15‐EEZE but not affected by TPPU. It has been demonstrated that 11,12‐EET promotes wound healing in ob/ob mice, while 14,15‐EEZE inhibits wound healing. Crucially, 14,15‐EEZE is primarily associated with the persistent or exacerbated inflammatory state observed in diabetic wounds (Zhao et al. 2017). Mechanistically, we also found that alterations in the Ephx2/11,12‐EET axis directly influenced pyroptosis at wound sites in diabetic mice, in the presence of the PNS. Directly, we can also infer that the anti‐inflammatory effects of the PNS would be blocked by 14,15‐EEZE. Based on these findings, we can speculate that Ephx2/11,12‐EET serves as a key mediator for the therapeutic effects of PNS.

Finally, we identified five major active saponin components in PNS through UPLC‐QE‐Orbitrap‐MS: R1, Rg1, Re, Rb1, and Rd. These saponins are also the most frequently reported bioactive compounds with anti‐inflammatory and antioxidant properties (Zeng et al. 2026). Among them, Rg1 has been reported to promote wound healing in diabetic conditions (Xiao et al. 2025). Molecular docking analysis revealed their high affinity for Ephx2, and these bioactivities were validated in vitro using macrophages, where they promoted the expression of 11,12‐EET. In vivo experiments demonstrate that these saponins, as key active components of PNS, exert similar pharmacological effects to PNS. These include inhibiting Ephx2, elevating 11,12‐EET, reducing inflammation and pyroptosis, and promoting wound healing. This suggests that the PNS efficacy is due to the combined action of these distinctive, high‐affinity saponins, functioning through a shared molecular target, rather than small impurities.

An important consideration for the clinical translation of our findings is the pharmacokinetic profile of orally administered PNS. It is well established that ginsenosides and notoginsenosides exhibit relatively low systemic bioavailability following oral administration, primarily due to their large molecular size, poor membrane permeability, and extensive intestinal metabolism by gut microbiota and hepatic enzymes (Guo et al. 2019). More importantly, in the model of diabetic rats, the oral bioavailability of Rb1 can be increased to 2.25%, while that of normal rats is only 0.90%, and that of high‐fat diet rats is 0.78% (Liu et al. 2015). The parent saponins are extensively deglycosylated by intestinal bacteria to yield secondary metabolites such as compound K, ginsenoside Rh2, and protopanaxadiol, which may exhibit greater systemic absorption and distinct pharmacological activities (Hasegawa 2004; Zhang et al. 2021). Therefore, the therapeutic effects observed in our study following oral PNS administration may be driven not only by the parent saponins (R1, Rg1, Re, Rb1, Rd) but also by their bioavailable microbial metabolites, which could reach the wound site via systemic circulation and exert anti‐inflammatory and mitochondrial‐protective effects. This hypothesis is consistent with the growing recognition that the gut microbiota plays a crucial role in mediating the systemic pharmacological effects of orally administered saponins (Zhao et al. 2023). Nevertheless, given the low oral bioavailability, topical or transdermal delivery of PNS, like wound dressings, hydrogels, or microneedle patches, may represent a more direct and efficient route for achieving therapeutic concentrations at the wound site, particularly for localized diabetic foot ulcers. Indeed, topical formulations of PNS and related saponins have shown promise in preclinical wound healing models (Lei et al. 2022; Men et al. 2020). Future studies should compare the pharmacokinetics and therapeutic efficacy of oral versus topical PNS administration and investigate the contribution of gut microbial metabolites to the systemic effects observed in the present study. Additionally, the development of nanoformulations or absorption enhancers to improve the oral bioavailability of PNS constituents warrants further investigation.

The limitations of this study are primarily reflected in three aspects. First, wound healing is a complex process involving multiple cell types (such as endothelial cells, keratinocytes, fibroblasts, etc.) and signaling pathways (such as VEGF, TGF‐β, Wnt, etc.). This study focused on macrophages, and future research could be expanded to include more cell types and signaling networks to comprehensively reveal the multi‐target repair mechanism of PNS. Second, the use of untargeted metabolomics for screening differential metabolites provides broad coverage, yet may overlook key metabolites with low abundance or those not indexed in databases. Third, while the inhibitors (TPPU, 14,15‐EEZE) used in in vitro and in vivo experiments exhibit some specificity, off‐target effects may still occur. Future functional validation could incorporate gene knockout or conditional knockout animal models.

## 5 Conclusion

This study reveals that PNS act as potent natural modulators by targeting the Ephx2/11,12‐EET pathway to stabilize mitochondrial homeostasis in macrophages, reduce pyroptosis, and thereby promote diabetic wound healing. These findings highlight the pharmacological potential of PNS as a candidate therapeutic agent for diabetic wounds and lay the groundwork for further clinical exploration of treatment strategies for diabetic foot ulcers.

## Author Contributions

**Nan Chen:** writing – original draft, data curation, investigation, methodology, software, funding acquisition. **Bo Zhu:** funding acquisition, data curation, formal analysis, visualization, project administration, supervision. **Cai‐hua Sun:** funding acquisition, investigation, supervision. **Tong Hou:** writing – original draft, validation, funding acquisition, formal analysis, data curation, conceptualization, methodology, resources, writing – review and editing, software.

## Funding

This study was supported by National Natural Science Foundation of China (82607093), Medical and Health Science Program of Zhejiang Province (2025HY0510), Zhejiang Provincial Traditional Chinese Medicine Science and Technology Plan Project (2026ZL0305), Zhejiang Chinese Medical University Young Talent Program (2023FSYYZQ02, 2024RCZXZK11), Renhe Outstanding Talent Program of Zhejiang Provincial Hospital of Traditional Chinese Medicine (2D12521), National Inheritance Studio for Veteran Chinese Pharmacy Practitioners (1X12602, 2B12648), Key Project at Central Government Level: The Ability Establishment of Sustainable Use for Valuable Chinese Medicine Resources (2060302).

## Ethics Statement

All experimental procedures were approved by the Laboratory Animal Management and Ethics Committee of Zhejiang Chinese Medical University (Approval No. IACUC‐20231030‐22) and were conducted in strict accordance with the National Institutes of Health Guide for the Care and Use of Laboratory Animals.

## Conflicts of Interest

The authors declare no conflicts of interest.

## Supporting information


**Figure S1:** Identification of RAW264.7 macrophages. (A) SSC‐A and FSC‐A circle the main cell populations. (B) FSC‐A and FSC‐H distinguish populations of individual cells. (C) Identification of macrophage purity using F4/80 and CD11b.
**Figure S2:** PNS improves mitochondrial ultrastructure. (A) TEM observations of mitochondrial ultrastructure in wound sections. The boxed region in the upper panel is magnified below. Red “N” indicates nucleus; yellow arrows indicate mitochondria. Scale bars: 1 μm (upper), 500 nm (lower). (B) The ratio of damaged mitochondria to total mitochondria was quantified. Values were expressed as means ± SEM, *n* = 8. **p < 0.01, vs. NW; ^#^ *p* < 0.05, vs. DW.
**Figure S3:** Untargeted metabolomics detects the molecular mechanism of PNS. (A) Quantitative values of 11,12‐DHET. (B)The ratio of 11,12‐EET/11,12‐DHET. *n* = 4. *p < 0.05, vs. NW; ^#^ *p* < 0.05, vs. DW.
**Figure S4:** PNS enhances the thermal stability of the Ephx2 protein. (A) The CETSA assay assessed the stability of the Ephx2 protein at different temperatures with PNS or PBS intervention. (B) Statistical graph of the CETSA experiment. Values were expressed as means ± SEM, *n* = 3. *p < 0.05, vs. PBS.
**Figure S5:** PNS promotes mitochondrial morphological integrity. (A) Relative statistics of mitochondrial length in MitoTracker staining; (B) qPCR detection of mRNA levels of Mfn1, Mfn2, OPA1, and Drp1. *n* = 6–8. **p < 0.01, vs. Con; ^#^ *p* < 0.05, ^##^ *p* < 0.01 vs. HL; ^&^ *p* < 0.05 vs. PNS.
**Figure S6:** Effects of five ginsenosides on Ephx2 mRNA in RAW264.7 cells. (A) 3D and 2D diagrams of R1, Rg1, Re, Rb1, and Rd. docked to the Ephx2 molecule, including binding energy and amino acid binding details. (B) qPCR analysis of Ephx2 mRNA in the RAW264.7 cells. (C) The 11,12‐EET level in the RAW264.7 cells was determined via ELISA. Values were expressed as means ± SEM, *n* = 6. ^#^ *p* < 0.05, ^##^ *p* < 0.01, vs. HL.
**Table S1:** Antibodies for Western blot.
**Table S2:** Primer sequences used for qPCR.
**Table S3:** Identification of *Panax notoginseng* saponins (PNS).

## Data Availability

The data that support the findings of this study are available from the corresponding author upon reasonable request.
