# Supplementary material for: Panax notoginseng Saponins Accelerate Diabetic Wound Repair by Minimizing Mitochondrial Dysfunction and Inhibiting Pyroptosis of Macrophage via Regulating Ephx2/11,12‐EET Axis
Source: Food Sci Nutr. 2026 Oct 16;14(10):e72494. doi: 10.1002/fsn3.72494 (PMC13675604; doi:10.1002/fsn3.72494)
Supplement: Supplementary file 1 [file FSN3-14-e72494-s001.docx]

# **1. Supplement Figures**

## Fig. S1

**Fig. S1. Identification of RAW264.7 macrophages.** (A) SSC-A and FSC-A circle the main cell populations. (B) FSC-A and FSC-H distinguish populations of individual cells. (C) Identification of macrophage purity using F4/80 and CD11b.

## Fig. S2

**Fig. S2. PNS improves mitochondrial ultrastructure.** (A) TEM observations of mitochondrial ultrastructure in wound sections. The boxed region in the upper panel is magnified below. Red “N” indicates nucleus; yellow arrows indicate mitochondria. Scale bars: 1 μm (upper), 500 nm (lower). (B) The ratio of damaged mitochondria to total mitochondria was quantified. Values were expressed as means ± SEM, *n* = 8. ** *p<* 0.01, vs. NW; # *p<* 0.05, vs. DW.

## Fig. S3

**Fig. S3. Untargeted metabolomics detects the molecular mechanism of PNS.** (A) Quantitative values of 11,12-DHET. (B)The ratio of 11,12-EET/11,12-DHET. *n* =4. * *p*< 0.05, vs. NW; # *p*< 0.05, vs. DW.

## Fig. S4

**Fig. S4. PNS enhances the thermal stability of the Ephx2 protein.** (A) The CETSA assay assessed the stability of the Ephx2 protein at different temperatures with PNS or PBS intervention. (B) Statistical graph of the CETSA experiment. Values were expressed as means ± SEM, *n* = 3. * *p<* 0.05, vs. PBS.

## Fig. S5

**Figure S5. PNS promotes mitochondrial morphological integrity.** (A) Relative statistics of mitochondrial length in MitoTracker staining; (B) qPCR detection of mRNA levels of Mfn1, Mfn2, OPA1, and Drp1. n = 6-8. ** *p*< 0.01, vs. Con; # *p*< 0.05, ## *p*< 0.01 vs. HL; & *p*< 0.05 vs. PNS.

## Fig. S6

**Fig. S6.** **Effects of five ginsenosides on Ephx2 mRNA in RAW264.7 cells.** (A,) 3D and 2D diagrams of R1, Rg1, Re, Rb1, and Rd docked to the Ephx2 molecule, including binding energy and amino acid binding details. (B) qPCR analysis of Ephx2 mRNA in the RAW264.7 cells. (C) The 11,12-EET level in the RAW264.7 cells was determined via ELISA. Values were expressed as means ± SEM, *n* = 6. # *p<* 0.05, ## *p<* 0.01, vs. HL.

# **2. Supplement Tables**

**Table S1.**

**Antibodies for Western blot**

| Gene | Primer | Sequences (5`-3`) |
| --- | --- | --- |
| iNOS | A0312 | ABclonal |
| Arg1 | A1847 | ABclonal |
| GSDMD | ab219800 | Abcam |
| Mfn1 | sc-166644 | Santa Cruz |
| Mfn2 | sc-100560 | Santa Cruz |
| OPA1 | 80471 | Cell Signaling Technology |
| p-Drp1 Ser616 | 4494 | Cell Signaling Technology |
| Drp1 | 8570 | Cell Signaling Technology |
| NLRP3 | ab270449 | Abcam |
| Pro-Caspase-1 | ab179515 | Abcam |
| Caspase-1 p20 | A0964 | ABclonal |
| β-actin | AC026 | ABclonal |
| VDAC1 | A19707 | ABclonal |

**Table S2.**

**Primer Sequences Used for qPCR**

| Gene | Primer | Sequences (5`-3`) |
| --- | --- | --- |
| Cyp2j5 | Forward | ATGGAAGGAGCAAAGGAGGC |
|  | Reverse | ATGGCTTCCACCAGGTGATG |
| Cyp2j6 | Forward | GGCCACTCCAGATGTGTTCA |
|  | Reverse | ATGAACAGCTCAGACCTGGC |
| Cyp2j9 | Forward | ATGCTTGCGGCAGCCGGCTC |
|  | Reverse | TCACCCCTCCATGCCAACTT |
| Ephx1 | Forward | TTGAAGGGCTGGACATCCAC |
|  | Reverse | ACACGTGCTCATCACTCAGG |
| Ephx2 | Forward  Reverse | ACCCTGAAGGCAAAACCCAA  TCCAGTTCTCTCAGAGCGGA |
| Mfn1 | Forward  Reverse | CTTGCTGTCATTGGGGAGGT  AGCAGTTGGTTGTGTGACCA |
| Mfn2 | Forward  Reverse | TGCCTGGATGCTGATGTGTT  TCCATGTACTCAGGCTCCGA |
| OPA1 | Forward  Reverse | TACTGTTAGCCCCGAGACCA  GATGACACCAGGCAAGTCCA |
| Drp1 | Forward  Reverse | GCCTCAGATCGTCGTAGTGG  GACCACACCAGTTCCTCTGG |
| β-actin | Forward  Reverse | CACTATTGGCAACGAGCGGTTC |
|  |  | GCATAGAGGTCTTTACGGATGTCAAC |

**Table S3.**

**Identification of Panax notoginseng Saponins (PNS)**

| **No.** | **Formula** | **Mass Error (ppm)** | **Metabolites** | **precursorion (m/z)** | **Retention time (min)** | **Ion mode** | **original square peak area** | **Proportion of original square peak area** |
| --- | --- | --- | --- | --- | --- | --- | --- | --- |
| M0001 | C5H11NO2 | 1.52431 | DL-Norvaline | 118.08643 | 0.752 | POS | 6120340.1 | 0.0680065 |
| M0002 | C10H14O | -4.057046 | 4-Isopropylbenzyl alcohol | 133.10123 | 23.326 | POS | 2743615 | 0.0304858 |
| M0003 | C10H14O | -3.906785 | Thymol | 133.10125 | 20.657 | POS | 22156350 | 0.2461914 |
| M0004 | C10H18O2 | -4.31012 | delta-Decalactone | 153.12732 | 26.523 | POS | 11612491 | 0.1290328 |
| M0005 | C12H14O4 | -2.57823 | Ethyl ferulic acid | 221.08136 | 23.49 | NEG | 2849850.7 | 0.0316663 |
| M0006 | C15H22O2 | -1.18901 | Curcumenol | 269.13106 | 27.88 | NEG | 76388784 | 0.848798 |
| M0007 | C16H32O3 | -0.884865 | 2-Hydroxypalmitic acid | 271.22763 | 33.901 | NEG | 4915863.4 | 0.0546229 |
| M0008 | C12H20O2 | -3.9633 | Bornyl acetate | 179.14292 | 22.979 | POS | 5257307.4 | 0.0584168 |
| M0009 | C12H22O12 | -2.296251 | Lactobionic acid | 357.10303 | 0.873 | NEG | 5674299.6 | 0.0630503 |
| M0010 | C6H12O6 | -0.984966 | Glucose | 203.05241 | 0.752 | POS | 228539258 | 2.5394259 |
| M0011 | C15H24O | -2.706959 | Spathulenol | 203.17947 | 31.186 | POS | 2025339.4 | 0.0225047 |
| M0012 | C10H13N5O3 | 0.237992 | Cordycepin | 252.10918 | 5.898 | POS | 5052829.7 | 0.0561448 |
| M0013 | C11H15N5O4 | -0.992487 | 2'-O-Methyladenosine | 282.1194 | 7.047 | POS | 8184539 | 0.0909429 |
| M0014 | C16H17N3O2 | -1.161402 | Brevianamide F | 284.13902 | 17.627 | POS | 771486.7 | 0.0085724 |
| M0015 | C18H34O2 | -1.731661 | Petroselinic acid | 300.28918 | 33.623 | POS | 1450667 | 0.0161192 |
| M0016 | C36H60O8 | 1.0219 | Ginsenoside Rh4 | 665.4277 | 28.664 | NEG | 501896992 | 5.5768547 |
| M0017 | C36H60O9 | 0.675059 | Ginsenoside Rh7 | 681.42239 | 23.7 | NEG | 5480996.7 | 0.0609024 |
| M0018 | C36H62O9 | 0.497485 | Ginsenoside F1 | 683.43792 | 24.382 | NEG | 217388956 | 2.4155288 |
| M0019 | C36H62O9 | -0.351166 | (20R)-Ginsenoside Rh1 | 683.43734 | 23.34 | NEG | 296966169 | 3.2997551 |
| M0020 | C16H28O7 | -1.154368 | Rosiridin | 355.17231 | 18.103 | POS | 1241663.1 | 0.0137968 |
| M0021 | C41H66O13 | 1.330953 | Ilexoside D | 811.44962 | 25.183 | NEG | 1540289.5 | 0.017115 |
| M0022 | C42H70O12 | 1.133724 | Ginsenoside Rg5 | 811.48585 | 31.006 | NEG | 120928595 | 1.3437044 |
| M0023 | C42H70O12 | 1.244632 | Ginsenoside F4 | 811.48594 | 28.117 | NEG | 36610548 | 0.4068 |
| M0024 | C42H70O12 | 0.813324 | Ginsenoside Rg6 | 811.48559 | 27.833 | NEG | 31823426 | 0.3536077 |
| M0025 | C41H70O13 | 0.429195 | Notoginsenoside R2 | 815.48019 | 22.34 | NEG | 306655663 | 3.4074205 |
| M0026 | C41H70O13 | 1.630942 | 20(R)-Notoginsenoside R2 | 815.48117 | 22.753 | NEG | 129161053 | 1.4351798 |
| M0027 | C12H22O11 | -2.081591 | Turanose | 365.10467 | 0.878 | POS | 173241753 | 1.9249848 |
| M0028 | C42H72O13 | 0.735387 | 20(R)-Ginsenoside Rg2 | 829.4961 | 22.753 | NEG | 534727796 | 5.9416559 |
| M0029 | C42H72O13 | 1.121163 | (20R)-Ginsenoside Rg3 | 829.49642 | 29.316 | NEG | 251422157 | 2.7936905 |
| M0030 | C42H72O13 | 0.614832 | Ginsenoside F2 | 829.496 | 28.7 | NEG | 92700837 | 1.0300502 |
| M0031 | C42H70O14 | 0.877323 | Bupleuroside XIII | 843.4755 | 21.358 | NEG | 9298356.8 | 0.1033192 |
| M0032 | C42H72O14 | 0.449443 | Pseudoginsenoside F11 | 845.49079 | 21.961 | NEG | 127131102 | 1.4126239 |
| M0033 | C21H26O6 | 7.503232 | Hexahydrocurcumin | 397.16514 | 21.516 | POS | 4394957 | 0.0488348 |
| M0034 | C47H80O17 | -0.790401 | Notoginsenoside Ft1 | 961.53699 | 28.7 | NEG | 5498405.6 | 0.0610958 |
| M0035 | C47H80O17 | 1.133601 | Notoginsenoside Fe | 961.53884 | 26.62 | NEG | 5511914.1 | 0.0612459 |
| M0036 | C47H80O17 | 0.728001 | Gynostemma Extract | 961.53845 | 27.253 | NEG | 25585634 | 0.2842961 |
| M0037 | C19H30O8 | -0.879801 | Roseoside | 409.18293 | 13.169 | POS | 2092131.3 | 0.0232468 |
| M0038 | C47H80O18 | 0.398964 | Notoginsenoside R1 | 977.53305 | 20.465 | NEG | 1.431E+09 | 15.901919 |
| M0039 | C48H82O20 | -0.419423 | Hosenkoside C | 977.53226 | 20.716 | NEG | 10951231 | 0.1216852 |
| M0040 | C48H80O18 | 0.949943 | 5,6-Dehydrogensenoside Rd | 989.5336 | 23.946 | NEG | 11493263 | 0.127708 |
| M0041 | C48H80O18 | 0.293068 | Nepasaikosaponin K | 989.53295 | 22.239 | NEG | 1695119.5 | 0.0188354 |
| M0042 | C48H82O18 | -0.121023 | Ginsenoside Re | 991.54819 | 20.606 | NEG | 346482684 | 3.8499605 |
| M0043 | C48H82O18 | 0.756393 | Gypenoside XVII | 991.54906 | 26.063 | NEG | 163489728 | 1.8166247 |
| M0044 | C30H48O2 | -2.362041 | Ganoderol B | 423.36173 | 35.426 | POS | 1430777.5 | 0.0158982 |
| M0045 | C48H80O19 | 0.596702 | 11-Oxomogroside III | 1005.5282 | 20.928 | NEG | 27183995 | 0.3020564 |
| M0046 | C48H82O19 | 0.416856 | Mogroside III | 1007.5437 | 20.351 | NEG | 158137593 | 1.7571542 |
| M0047 | C30H48O | -1.481036 | Lupenone | 425.37716 | 22.358 | POS | 510961028 | 5.6775702 |
| M0048 | C54H92O23 | -3.241255 | Ginsenoside Rb1 | 1107.5921 | 22.398 | NEG | 366308787 | 4.0702593 |
| M0049 | C53H90O22 | -0.409402 | Ginsenoside Rc | 1123.5901 | 22.81 | NEG | 6271721 | 0.0696886 |
| M0050 | C53H90O22 | -0.195801 | Ginsenoside Rb3 | 1123.5904 | 23.302 | NEG | 93722013 | 1.0413971 |
| M0051 | C30H52O3 | -2.34557 | (20S)-Protopanaxadiol | 443.38791 | 35.013 | POS | 1665751.2 | 0.0185091 |
| M0052 | C54H92O24 | -0.367648 | Mogroside IV | 1169.5956 | 20.716 | NEG | 10222291 | 0.1135855 |
| M0053 | C30H48O4 | -2.39375 | 23-Hydroxybetulinic acid | 455.35147 | 30.569 | POS | 2915371 | 0.0323943 |
| M0054 | C30H52O4 | -2.046219 | (20S)-Protopanaxatriol | 459.38292 | 29.994 | POS | 7790452.1 | 0.086564 |
| M0055 | C30H52O4 | -2.394512 | Mogrol | 459.38276 | 28.66 | POS | 1340886.2 | 0.0148993 |
| M0056 | C30H52O4 | -0.314206 | Panaxatriol | 477.39369 | 30.836 | POS | 705608.42 | 0.0078404 |
| M0057 | C30H46O4 | -0.222975 | 18beta-Glycyrrhetinic acid | 493.32872 | 32.846 | POS | 2459854.1 | 0.0273328 |
| M0058 | C30H52O5 | -0.668844 | 20(S),24(R)-Ocotillol | 493.38842 | 28.66 | POS | 1854519.2 | 0.0206066 |
| M0059 | C30H48O4 | 0.080752 | Alisol B | 495.34452 | 33.168 | POS | 16914129 | 0.1879422 |
| M0060 | C18H32O16 | -1.498601 | Manninotriose | 527.15746 | 0.884 | POS | 4923169.3 | 0.0547041 |
| M0061 | C30H48O6 | -0.663716 | 19alpha-Hydroxyasiatic acid | 527.33396 | 23.666 | POS | 3347118.6 | 0.0371917 |
| M0062 | C30H50O6 | -0.245584 | Gymnemagenin | 529.34983 | 21.496 | POS | 1448361.3 | 0.0160935 |
| M0063 | C36H60O8 | -0.944606 | Ginsenoside Rk3 | 603.42555 | 28.359 | POS | 3840345.9 | 0.0426722 |
| M0064 | C36H62O8 | -0.545057 | 20(R)-Ginsenoside Rh2 | 605.44144 | 31.711 | POS | 1164835.8 | 0.0129431 |
| M0065 | C36H62O8 | -0.677191 | Ginsenoside Rh2 | 605.44136 | 31.186 | POS | 1719934.1 | 0.0191111 |
| M0066 | C36H62O9 | -1.303431 | Ginsenoside Rh1 | 621.43587 | 22.979 | POS | 96561216 | 1.072945 |
| M0067 | C36H62O10 | -0.228853 | Pseudoginsenoside RT5 | 655.44142 | 22.317 | POS | 7284441.7 | 0.0809415 |
| M0068 | C36H58O10 | 0.460356 | Arjunetin | 673.39253 | 21.756 | POS | 9186597.6 | 0.1020774 |
| M0069 | C42H72O14 | -0.382902 | Mogroside IIe | 783.48921 | 21.262 | POS | 8134984.7 | 0.0903923 |
| M0070 | C42H72O13 | 0.050923 | Gypenoside LXXV | 785.50461 | 29.494 | POS | 4894655.4 | 0.0543872 |
| M0071 | C42H72O14 | 0.437168 | Ginsenoside Rg1 | 823.48178 | 20.677 | POS | 342724182 | 3.8081977 |
| M0072 | C48H82O18 | -0.527138 | 11-Deoxymogroside IIIE | 929.54693 | 22.358 | POS | 41766217 | 0.4640875 |
| M0073 | C47H80O17 | 0.535003 | Ginsenoside Rd2 | 934.57387 | 27.344 | POS | 2000851.1 | 0.0222326 |
| M0074 | C48H82O18 | -0.350682 | Ginsenoside Rd | 969.53899 | 24.701 | POS | 229938235 | 2.5549707 |
| M0075 | C48H82O19 | 0.497192 | Mogroside IIIA2 | 985.53474 | 20.371 | POS | 28472187 | 0.3163702 |
| M0076 | C54H92O24 | 0.48798 | Mogroside IVe | 1147.5876 | 20.737 | POS | 5861163.1 | 0.0651266 |
| M0077 | C58H98O26 | 0.1465 | GinsenosideRa1 | 1228.6686 | 22.692 | POS | 23269256 | 0.2585576 |
| M0078 | C59H100O27 | 2.053715 | Ginsenoside Ra3 | 1241.655 | 22.255 | POS | 5793760.6 | 0.0643777 |
| M0079 | C59H100O27 | -0.617267 | Notoginsenoside Fa | 1263.6336 | 21.516 | POS | 17155219 | 0.1906211 |
| M0080 | C4H9NO2 | 3.747456 | NSC 16590 | 104.07099 | 0.782 | POS | 857271.67 | 0.0095256 |
| M0081 | C9H10O | -3.843839 | 4-Ethylbenzaldehyde | 117.07001 | 24.68 | POS | 1417891 | 0.015755 |
| M0082 | C9H10O | -3.587583 | Cinnamyl Alcohol | 117.07004 | 20.657 | POS | 3384136.4 | 0.037603 |
| M0083 | C9H10O | -3.673002 | 4'-Methylacetophenone | 117.07003 | 22.337 | POS | 2212763.1 | 0.0245872 |
| M0084 | C10H14O | -4.357568 | Verbenone | 133.10119 | 22.337 | POS | 7829079 | 0.0869932 |
| M0085 | C10H16O | -4.514592 | Perillyl alcohol | 135.11681 | 24.68 | POS | 5588377.5 | 0.0620955 |
| M0086 | C7H13NO2 | -0.208186 | Stachydrine | 144.10187 | 0.762 | POS | 1293707.2 | 0.0143751 |
| M0087 | C7H13NO | -0.206706 | Nortropine | 145.13351 | 0.601 | POS | 695539.43 | 0.0077285 |
| M0088 | C11H18O | -4.425578 | Dihydrojasmone | 149.13241 | 28.704 | POS | 1657587.7 | 0.0184184 |
| M0089 | C8H7NO | 0 | 5-Hydroxyindole | 151.08659 | 9.631 | POS | 1755981.6 | 0.0195117 |
| M0090 | C10H14O | -0.066176 | Umbellulone | 151.11173 | 24.083 | POS | 3941574.5 | 0.043797 |
| M0091 | C7H6O4 | 1.032032 | 3,4,5-Trihydroxybenzaldehyde | 155.03404 | 6.579 | POS | 23485.59 | 0.000261 |
| M0092 | C8H15NO | -0.502673 | Tropine | 159.14911 | 0.622 | POS | 721604.56 | 0.0080181 |
| M0093 | C6H13NO4 | -0.365649 | 1-Deoxymannojirimycin | 164.09167 | 0.742 | POS | 2257274.3 | 0.0250818 |
| M0094 | C9H8O2 | 0.180629 | Cinnamic acid | 166.08628 | 20.472 | POS | 3853575.8 | 0.0428192 |
| M0095 | C14H20O7 | -1.805334 | Salidroside | 299.11309 | 10.078 | NEG | 3057.13 | 3.397E-05 |
| M0096 | C9H9NO2 | -0.220876 | 4-Aminocinnamic Acid | 181.09711 | 9.711 | POS | 909230.01 | 0.010103 |
| M0097 | C9H11NO2 | -0.764556 | Polygonatine A | 183.11266 | 9.405 | POS | 1359013.4 | 0.0151007 |
| M0098 | C18H36O4 | -0.69785 | 9,10-Dihydroxystearic acid | 315.25386 | 30.524 | NEG | 491905.19 | 0.0054658 |
| M0099 | C6H12O5 | -1.764162 | (-)-Fucose | 187.05736 | 0.878 | POS | 743459.77 | 0.008261 |
| M0100 | C9H21N2O2+ | -0.475788 | Laminine | 189.15966 | 0.642 | POS | 5470110.2 | 0.0607814 |
| M0101 | C14H18O9 | -2.218253 | Vanillic acid 4-beta-D-glucopyranoside | 329.08708 | 8.399 | NEG | 515620.03 | 0.0057293 |
| M0102 | C14H20O6 | -1.336881 | Phenylethyl-beta-D-galactoside | 329.12375 | 14.744 | NEG | 530352.15 | 0.005893 |
| M0103 | C21H18O6 | -1.670761 | Neoglycyrol | 365.10245 | 29.399 | NEG | 26190.71 | 0.000291 |
| M0104 | C16H26O7 | -1.785876 | Picrocrocin | 375.16538 | 14.304 | NEG | 2202464.4 | 0.0244728 |
| M0105 | C17H26O11 | -0.335805 | Shanzhiside methyl ester | 387.12899 | 11.708 | NEG | 330568.35 | 0.0036731 |
| M0106 | C22H26N2O3 | 1.371011 | Hirsuteine | 401.16429 | 21.844 | NEG | 827937.89 | 0.0091997 |
| M0107 | C14H12O2 | 0.094527 | Pinosylvin | 423.16022 | 24.184 | NEG | 285500.02 | 0.0031723 |
| M0108 | C11H16O4 | -0.765651 | Dihydrosinapyl alcohol | 235.0939 | 15.442 | POS | 910368.31 | 0.0101156 |
| M0109 | C17H16O2 | -0.671612 | Effusol | 253.12214 | 25.225 | POS | 4631.44 | 5.146E-05 |
| M0110 | C21H36O10 | -1.094826 | A-D-Glucopyranoside | 493.22851 | 21.143 | NEG | 787781.3 | 0.0087535 |
| M0111 | C18H32O16 | 0.079497 | 1-Kestose | 503.1618 | 1.339 | NEG | 48679.57 | 0.0005409 |
| M0112 | C12H16O6 | -0.179158 | Phenyl β-D-glucopyranoside | 279.08386 | 9.74 | POS | 374976.04 | 0.0041666 |
| M0113 | C15H22O3 | 3.597122 | 5α-Hydroxycostic acid | 289.12109 | 28.581 | POS | 458404.8 | 0.0050936 |
| M0114 | C10H18O9 | -1.704447 | Xylobiose | 305.08378 | 0.878 | POS | 729997.68 | 0.0081114 |
| M0115 | C36H60O7 | 1.231845 | Ginsenoside Rh3 | 649.4329 | 33.433 | NEG | 4790998.7 | 0.0532354 |
| M0116 | C36H58O11 | 0.751438 | Arjunglucoside I | 665.39114 | 20.949 | NEG | 5066022.6 | 0.0562914 |
| M0117 | C36H62O8 | 0.928919 | Pseudoginsenoside RH2 | 667.44329 | 30.149 | NEG | 277408.11 | 0.0030824 |
| M0118 | C11H15N5O5 | -0.687293 | N2-Methylguanosine | 320.09632 | 7.112 | POS | 231833.01 | 0.002576 |
| M0119 | C36H58O10 | 1.308597 | Rosamultin | 695.40211 | 22.121 | NEG | 95608.63 | 0.0010624 |
| M0120 | C18H26O5 | -1.158859 | Beta-Zearalanol | 345.16684 | 23.243 | POS | 1134705.9 | 0.0126083 |
| M0121 | C20H22N2O3 | 2.076686 | Picrinine | 361.15301 | 20.121 | POS | 7685715 | 0.0854002 |
| M0122 | C42H72O15 | 1.017806 | Momordicoside A | 815.48068 | 21.121 | NEG | 120641880 | 1.3405186 |
| M0123 | C16H22O8 | -1.424187 | Coniferin | 365.12017 | 11.024 | POS | 681789.86 | 0.0075757 |
| M0124 | C43H70O15 | 1.5991 | Isoastragaloside II | 825.46552 | 26.982 | NEG | 841318.61 | 0.0093484 |
| M0125 | C42H70O13 | 2.042346 | Damulin B | 827.48153 | 29.593 | NEG | 1141197.8 | 0.0126805 |
| M0126 | C23H32O5 | -2.693804 | Ingenol-5,20-acetonide | 371.22127 | 28.66 | POS | 775404.8 | 0.008616 |
| M0127 | C45H73NO15 | -0.438551 | α-Solanine | 866.49036 | 20.655 | NEG | 3309945.5 | 0.0367786 |
| M0128 | C16H22O11 | -0.920425 | beta-D-Glucose pentaacetate | 391.12313 | 22.067 | POS | 1857065.9 | 0.0206349 |
| M0129 | C17H24O9 | -1.16417 | Syringin | 395.13079 | 11.924 | POS | 331513.86 | 0.0036836 |
| M0130 | C49H72O17 | -0.021895 | Qingyangshengenin A | 913.45854 | 29.316 | NEG | 710082.13 | 0.0078901 |
| M0131 | C48H82O19 | 0.4264 | Mogroside III-A1 | 961.53816 | 21.058 | NEG | 5149196.4 | 0.0572156 |
| M0132 | C24H40O4 | -0.505681 | Hyodeoxycholic acid | 415.28167 | 27.984 | POS | 744412.9 | 0.0082716 |
| M0133 | C30H48O3 | -2.139487 | Gadermandiol | 439.35671 | 34.488 | POS | 458759.86 | 0.0050975 |
| M0134 | C22H26O8 | -0.929385 | Eupalinolide O | 441.15158 | 22.234 | POS | 5956188.1 | 0.0661825 |
| M0135 | C54H92O25 | -0.39488 | Hosenkoside K | 1139.585 | 20.351 | NEG | 5316875.1 | 0.0590787 |
| M0136 | C30H50O4 | -2.033373 | Bryodulcosigenin | 457.36728 | 29.582 | POS | 1168754 | 0.0129867 |
| M0137 | C30H50O4 | -1.858459 | Soyasapogenol A | 457.36736 | 30.846 | POS | 1550834.2 | 0.0172322 |
| M0138 | C30H48O5 | -0.551744 | Asiatic acid | 489.35718 | 28.123 | POS | 1820479.2 | 0.0202283 |
| M0139 | C24H30O11 | -3.412858 | 6'-O-cinnamoyl harpagide | 495.1844 | 20.453 | POS | 2534772.5 | 0.0281653 |
| M0140 | C30H46O5 | 0.314142 | Alisol C | 509.3239 | 27.752 | POS | 2817398.9 | 0.0313057 |
| M0141 | C27H41NO7 | 2.04193 | Funiculosin | 509.32317 | 30.436 | POS | 310477.9 | 0.0034499 |
| M0142 | C42H72O13 | -0.912059 | Ginsenoside Rg2 | 767.49389 | 22.358 | POS | 106161953 | 1.1796241 |
| M0143 | C42H68O13 | 0.676268 | Saikosaponin D | 798.50036 | 29.102 | POS | 180240.01 | 0.0020027 |
| M0144 | C42H72O14 | -0.124766 | Gypenoside L | 801.49938 | 28.123 | POS | 2313778.9 | 0.0257097 |
| M0145 | C42H70O14 | 0.754748 | 11-Oxomogroside IIe | 821.46639 | 20.863 | POS | 3253428 | 0.0361506 |
| M0146 | C42H72O14 | -0.226337 | Mogroside IIA1 | 839.45517 | 21.963 | POS | 2012019.3 | 0.0223567 |
